# Anti-TNFR2 Antibody-Conjugated PLGA Nanoparticles for Targeted Delivery of Adriamycin in Mouse Colon Cancer

**DOI:** 10.34133/research.0444

**Published:** 2024-09-06

**Authors:** Ping Li, Yang Yang, Yifei Wang, Jingbin Zheng, Fengyang Chen, Mengmeng Jiang, Chon-kit Chou, Weihong Cong, Zongjin Li, Xin Chen

**Affiliations:** ^1^ Institute of Chinese Medical Sciences, State Key Laboratory of Quality Research in Chinese Medicine, University of Macau, Macau, China.; ^2^ Center for Cancer Immunology, Institute of Biomedicine and Biotechnology, Shenzhen Institutes of Advanced Technology, Chinese Academy of Sciences, Shenzhen, China.; ^3^ Laboratory of Cardiovascular Diseases, Xiyuan Hospital of China Academy of Chinese Medical Sciences, Beijing, China.; ^4^ Faculty of Innovation Engineering, Macau University of Science and Technology, Macau, China.; ^5^ Department of Pharmaceutical Sciences, Faculty of Health Sciences, University of Macau, Macau, China.; ^6^ MoE Frontiers Science Center for Precision Oncology, University of Macau, Macau, China.

## Abstract

High levels of tumor necrosis factor receptor type II (TNFR2) are preferentially expressed by immunosuppressive CD4^+^Foxp3^+^ regulatory T cells (T_regs_), especially those present in the tumor microenvironment, as initially reported by us. There is compelling evidence that targeting TNFR2 markedly enhances antitumor immune responses. Furthermore, a broad spectrum of human cancers also expresses TNFR2, while its expression by normal tissue is very limited. We thus hypothesized that TNFR2 may be harnessed for tumor-targeted delivery of chemotherapeutic agents. In this study, we performed a proof-of-concept study by constructing a TNFR2-targeted PEGylated poly(dl-lactic-co-glycolic acid) (PLGA-PEG) nanodrug delivery system [designated as TNFR2-PLGA-ADR (Adriamycin)]. The results of in vitro study showed that this TNFR2-targeted delivery system had the properties in cellular binding and cytotoxicity toward mouse colon cancer cells. Further, upon intravenous injection, TNFR2-PLGA-ADR could efficiently accumulate in MC38 and CT26 mouse colon tumor tissues and preferentially bind with tumor-infiltrating T_regs_. Compared with ADR and ISO-PLGA-ADR, the in vivo antitumor effect of TNFR2-PLGA-ADR was markedly enhanced, which was associated with a decrease of TNFR2^+^ T_regs_ and an increase of IFNγ^+^CD8^+^ cytotoxic T lymphocytes in the tumor tissue. Therefore, our results clearly show that targeting TNFR2 is a promising strategy for designing tumor-specific chemoimmunotherapeutic agent delivery system.

## Introduction

Adriamycin (ADR), also known as doxorubicin, is clinically used for the treatment of sarcomas, lymphomas, and various carcinomas, including breast and bladder cancer [1,2]. Although ADR exhibits marked anticancer activity as single agents, its use in therapy has been complicated by severe side effects and toxicity that occur during or after treatment, including cardiotoxicity [3]. Dose-limiting toxicity of chemotherapeutics, due to their poor specificity, is a major limitation of their clinical application [4]. The therapeutic index of chemotherapeutics can be enhanced by reduction of systemic toxicity through targeted delivery [5]. To date, antibody–drug conjugates (ADCs) are the most successful targeted delivery systems, some of which have been approved by the United States Food and Drug Administration (US FDA) [6]. However, the translation of ADCs into clinically useful therapeutic options is still hampered by their structure and the emergence of resistance mechanisms [7]. In contrast to ADCs, antibody-conjugated nanoparticles hold promise to deliver drugs in a controlled manner while preserving their chemical structure, avoiding unpredicted metabolization, and reducing systemic toxicity [8]. Recently, antibody-conjugated nanoparticles, such as PD-L1, epidermal growth factor receptor (EGFR), and HER2, were developed for the cancer treatments [9].

TNFR2 is one of the receptors that mediate the biological function of tumor necrosis factor-α (TNF-α) [10,11]. We for the first time found that TNFR2 plays a decisive role in the activation, expansion, in vivo function, and phenotypical stability of CD4^+^Foxp3^+^ regulatory T cells (T_regs_) [11,12]. Further, we found that TNFR2 expression defines the maximally suppressive subset of T_regs_, and T_regs_ present in the tumor microenvironment characteristically express high levels of TNFR2 [13]. Since T_regs_ represent major cellular mechanism of tumor immune evasion [14,15], elimination of T_reg_ activity by targeting TNFR2 has become a strategy in the cancer immunotherapy [16,17]. Recently, it was reported that aberrant high levels of TNFR2 were expressed by a broad spectrum of human tumors, including ovarian cancer, colorectal cancer, and renal cell carcinoma [18,19]. Furthermore, expression of TNFR2 on the tumor cells promotes the growth of colon cancer, ovarian cancer, and multiple myeloma [20–22]. We recently showed that genetical ablation of TNFR2 impaired the growth of mouse tumor in both in vitro and in vivo settings [23]. Therefore, TNFR2 expressed on the surface of tumor cells and tumor-infiltrating T_regs_ may provide an opportunity for device of receptor-targeted cancer treatment [17]. This possibility was examined by our present proof-of-concept study. To this end, we encapsulated ADR with PEGylated poly(dl-lactic-co-glycolic acid) (PLGA-PEG) (designated as PLGA-ADR), and then PLGA-ADR nanoparticles were modified with anti-TNFR2 antibodies or control IgG (designated as TNFR2-PLGA-ADR and ISO-PLGA-ADR, respectively). The results showed that TNFR2 conditioning could markedly enhance the binding of nanoparticles to TNFR2-expressing T_regs_ and tumor cells. More importantly, in mouse MC38 and CT26 colon cancer models, treatment with TNFR2-PLGA-ADR potently inhibited the growth of tumor, which is associated with a decrease of TNFR2^+^ T_regs_ and, consequently, an increase of IFNγ^+^CD8^+^ cytotoxic T lymphocytes (CTLs) in the tumor tissue. Therefore, our study provides novel experiment evidence for the first time that targeting TNFR2 is a promising strategy to device antibody–nanodrug conjugated immunochemotherapeutic agent for tumor-specific delivery.

## Results

### Preparation and characterization of nanoparticles

PLGA-ADR nanoparticles were prepared by using a modified procedure of oil-in-water single-emulsion solvent evaporation as described previously [24,25]. In brief, ADR was dissolved in methanol, and PLGA-PEG-NHS (*N*-hydroxysuccinimide) was dissolved in tetrahydrofuran. Then, ADR and PLGA-PEG-NHS were mixed and quickly injected into an aqueous solution containing 6.25% methanol under the condition of intense ultrasound. After organic solvent was volatilized and free ADR was removed, PLGA-ADR nanoparticles were obtained. Then, isotype immunoglobulin G (IgG) or anti-TNFR2 antibody was conjugated onto the surface of PLGA-ADR nanoparticles by 1-ethyl-3-(3-dimethylaminopropyl)carbodiimide (EDC)/NHS chemistry to obtain ISO-PLGA-ADR and TNFR2-PLGA-ADR nanoparticles.

**Fig. 1.**
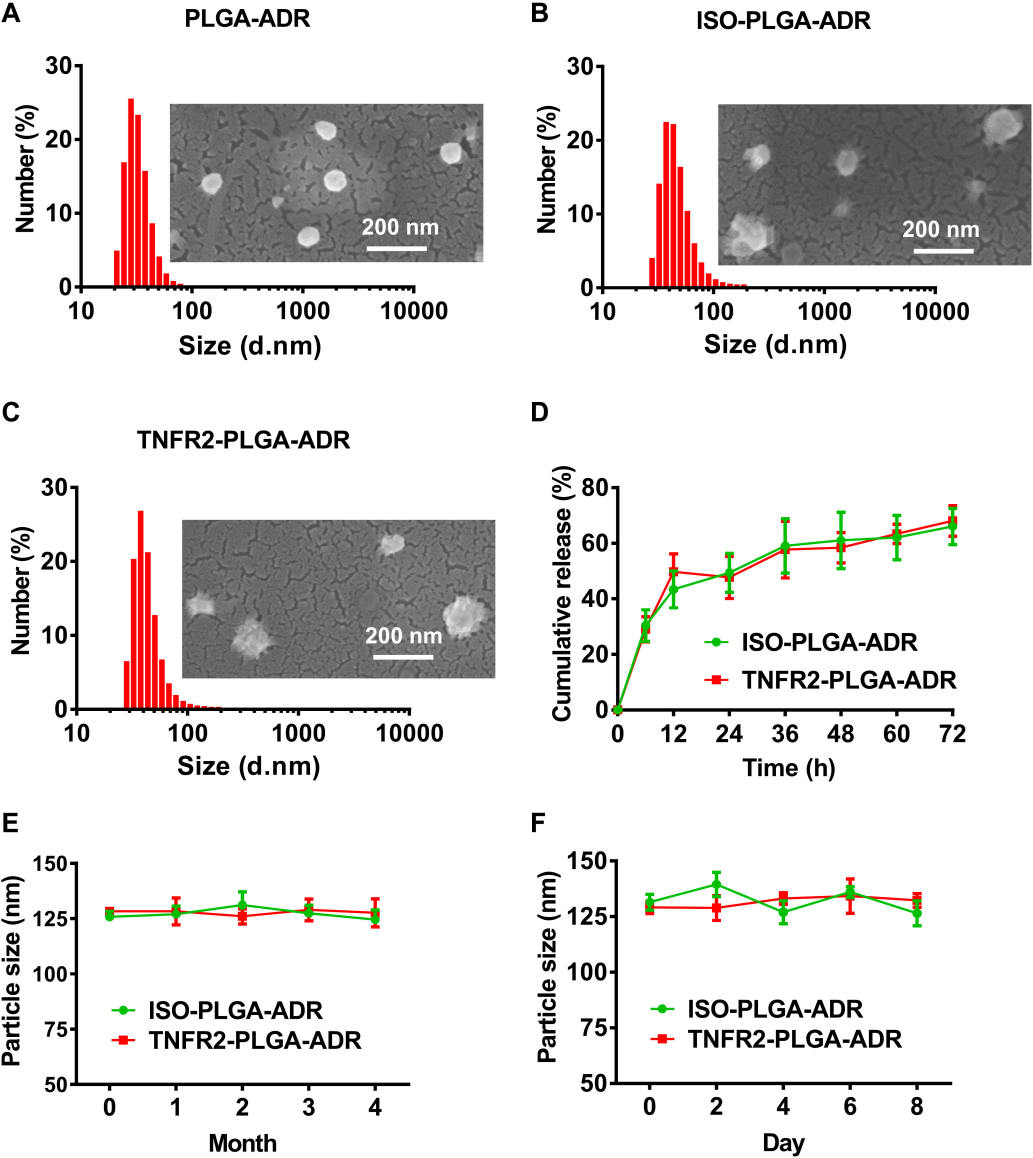
Characterization of PLGA-ADR, ISO-PLGA-ADR, and TNFR2-PLGA-ADR nanoparticles. (A to C) Size distribution and scanning electron microscopy image of PLGA-ADR (A), ISO-PLGA-ADR (B), and TNFR2-PLGA-ADR (C) nanoparticles. Scale bar, 200 nm. (D) In vitro release curves of ADR from ISO-PLGA-ADR and TNFR2-PLGA-ADR nanoparticles in PBS at pH 7.4 (*n* = 3). (E) Particle size of ISO-PLGA-ADR and TNFR2-PLGA-ADR nanoparticles at 4°C for different periods determined by DLS (*n* = 3). (F) Size of ISO-PLGA-ADR and TNFR2-PLGA-ADR nanoparticles in PBS (pH 7.4) containing 10% FBS at 37°C for different periods determined by DLS (*n* = 3).

The sizes of PLGA-ADR, ISO-PLGA-ADR, and TNFR2-PLGA-ADR nanoparticles, determined by a dynamic laser scattering (DLS) method, were 88.4 ± 1.33 nm, 125.8 ± 1.94 nm, and 128.1 ± 2.02 nm in diameter with a narrow distribution [PDI (polydispersity index) = 0.227 ± 0.003, 0.248 ± 0.006, and 0.251 ± 0.011] (Table), respectively. These nanoparticles were generally spherical, and the particle size determined by scanning electron microscopy was consistent with that determined by DLS (Fig. 1A to C). The ζ potential of these 3 nanoparticles was −0.15 ± 0.012, −11.7 ± 0.021, and −11.9 ± 0.018, respectively (Table). The encapsulation efficiency (EE) and loading capacity (LC) of free ADR within polymeric PLGA-ADR nanoparticles were 55.16 ± 0.43% and 5.68 ± 0.014% (w/w) (Table). The amount of anti-TNFR2 antibody (or isotype control antibody) conjugated to the PLGA-ADR surface was 10 μg per injection, as determined by a BCA (bicinchoninic acid) assay kit.

**Table.** Characterizations of the different PLGA nanoparticles

PLGA nanoparticles	Size (nm)	Polydispersity index	Zeta potential (mV)	Encapsulation efficiency %
PLGA-ADR	88.4 ± 1.33	0.227 ± 0.003	−0.15 ± 0.012	55.16 ± 0.43
ISO-PLGA-ADR	125.8 ± 1.94	0.249 ± 0.006	−11.7 ± 0.021	
TNFR2-PLGA-ADR	128.1 ± 2.02	0.251 ± 0.011	−11.9 ± 0.018	

To mimic the release of drug from nanoparticles before reaching the tumor site, in vitro drug release profile of nanoparticles was examined during 72-h period. A burst release of 43% ADR from nanoparticles was observed in the first 12 h. Afterward, a steady sustained release was observed during the following 60 h, which is desirable for a sustainable drug concentration in the tumor environment (Fig. 1D). Given that the ISO-PLGA-ADR and TNFR2-PLGA-ADR nanoparticles were stored in water but administered in phosphate-buffered saline (PBS) for in vivo experiments, we conducted long-term stability tests in water and short-term stability tests in PBS with 10% fetal bovine serum (FBS) to ensure their integrity and performance under respective conditions. The hydrodynamic size of ISO-PLGA-ADR and TNFR2-PLGA-ADR nanoparticles did not significantly change during the 4-month storage period in double-distilled water at 4°C and 8-d storage in PBS with 10% FBS, indicating satisfying stability (Fig. 1E and F).

### TNFR2 conditioning enhances the cytotoxicity of ADR-encapsulated nanoparticles

**Fig. 2.**
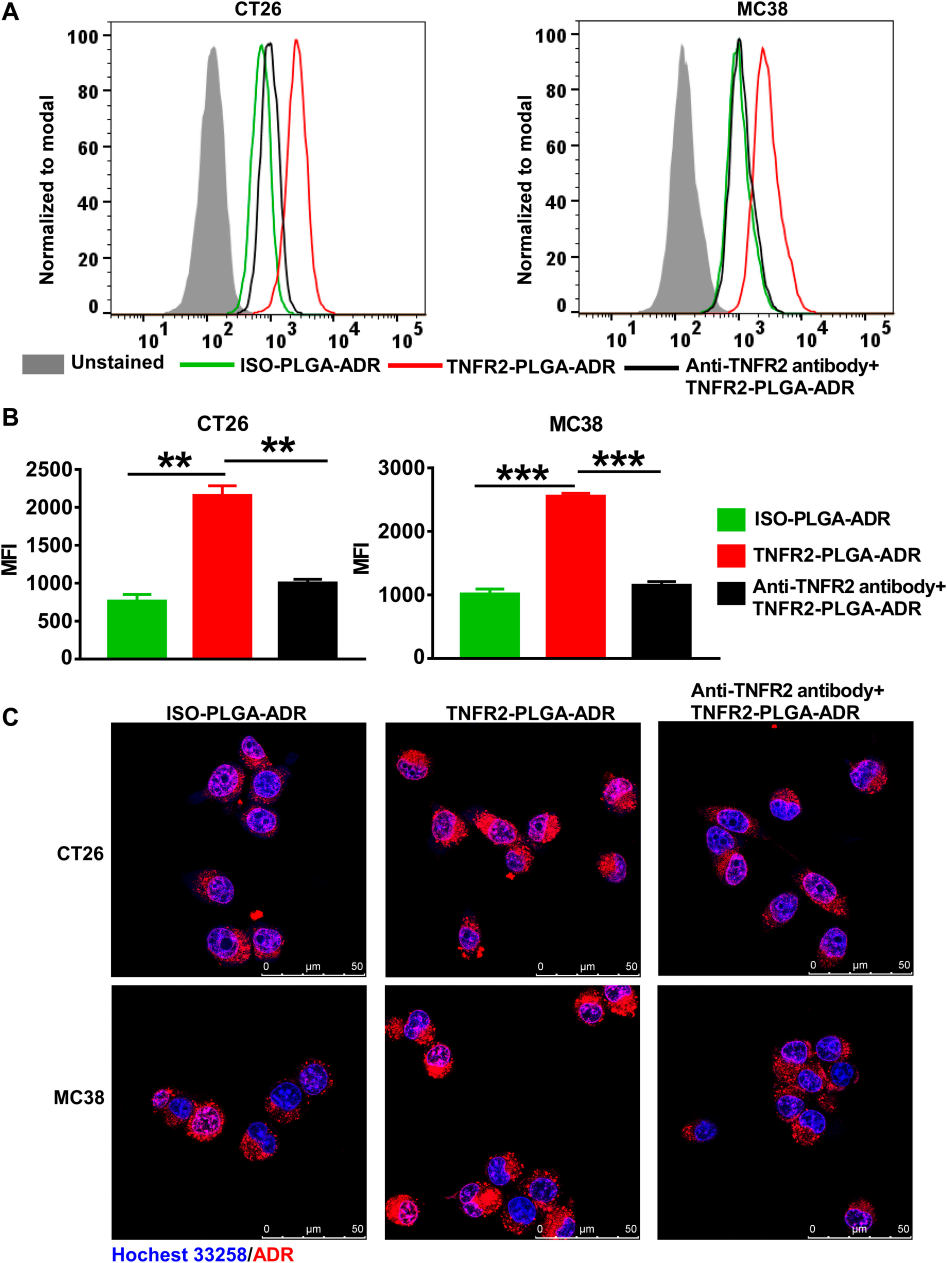
Binding of ISO-PLGA-ADR and TNFR2-PLGA-ADR nanoparticles with CT26 and MC38 colon cancer cells. CT26 and MC38 cells were incubated at 37°C for 4 h with ISO-PLGA-ADR, TNFR2-PLGA-ADR, or TNFR2-PLGA-ADR plus anti-TNFR2 antibody (25 μg/ml) pretreated for 1 h (equivalent ADR concentration of 1 μg/ml). The cellular binding was determined by the expression of ADR by using flow cytometry. Representative FACS plots (A) and summary of mean fluorescence intensity [MFI; (B)] are shown. (C) CT26 and MC38 cells were incubated at 37°C for 4 h with ISO-PLGA-ADR, TNFR2-PLGA-ADR, or TNFR2-PLGA-ADR plus anti-TNFR2 antibody (25 μg/ml) pretreated for 1 h, followed by staining with Hoechst 33258 (blue). The intracellular and nuclear accumulation of ADR (red) was determined and photographed with a confocal microscope. Data [means ± SEM (standard error of the mean), *n* = 3] shown are representatives of 3 separate experiments with similar results. By comparison with the indicated group, **P < 0.01, ***P < 0.001.

TNFR2 was expressed by mouse MC38 and CT26 tumor cells (Fig. S2). We thus used these 2 cell lines to examine the ability of nanoparticles conditioned with anti-TNFR2 antibody to bind with cancer cells. To this end, TNFR2-expressing mouse MC38 or CT26 tumor cells were treated with ISO-PLGA-ADR or TNFR2-PLGA-ADR nanoparticles for 4 h. Then, the proportion of ADR^+^ cells was analyzed by fluorescence-activated cell sorting (FACS) and confocal laser scanning microscopy (CLSM). As shown in Fig. 2, the level of ADR associated with cells was markedly enhanced in cells treated with TNFR2-PLGA-ADR (Fig. 2A to C; *P* < 0.001 to 0.01). The elevated binding of nanoparticle to the cells was mediated by TNFR2, since it could be almost completely abrogated by anti-TNFR2 antibody (Fig. 2A to C; *P* < 0.001 to 0.01).

**Fig. 3.**
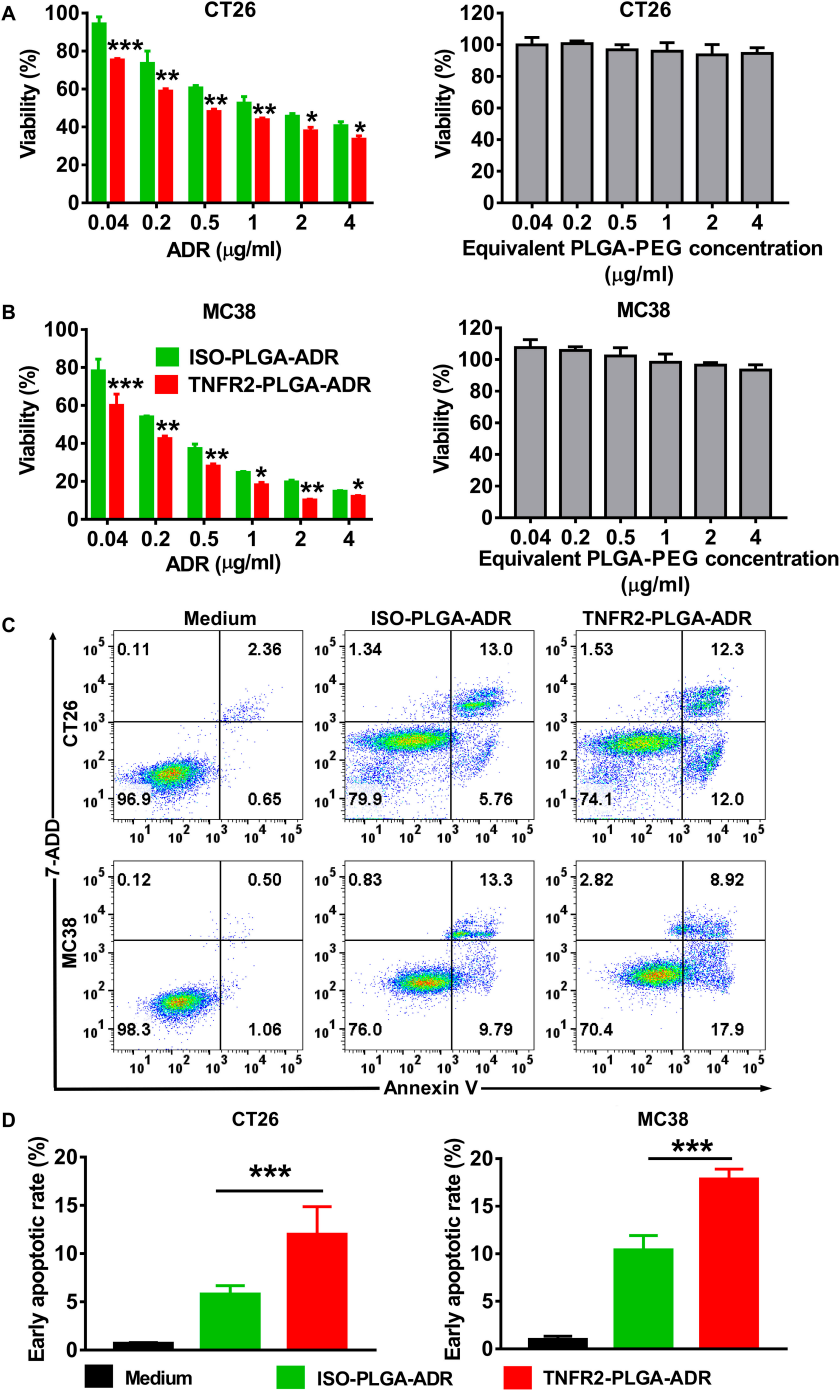
The cytotoxic effects of ISO-PLGA-ADR and TNFR2-PLGA-ADR nanoparticles on CT26 and MC38 tumor cells. (A and B) Cell viability of CT26 (A) and MC38 (B) cells was examined by MTT assay after treatment with PLGA-PEG, ISO-PLGA-ADR, and TNFR2-PLGA-ADR nanoparticles at different concentrations for 24 h. (C and D) Apoptosis of CT26 and MC38 cells analyzed by FACS after treatments with ISO-PLGA-ADR and TNFR2-PLGA-ADR nanoparticles (ADR: 1 μg/ml) for 24 h. The typical FACS plots (C) and summary of the proportion of apoptotic cells in each group (D). Data (means ± SEM, *n* = 5) shown are representatives of 3 separate experiments with similar results. By comparison with the ISO-PLGA-ADR group, *P < 0.05, **P < 0.01, ***P < 0.001.

We thus further examined the effect of TNFR2-PLGA-ADR and ISO-PLGA-ADR on the death of CT26 and MC38 cells. The cells were treated with nanoparticles in a concentration range of ADR from 0.04 to 4 μg/ml for 24 h. The results showed that both nanoparticles potently inhibited the viability of CT26 and MC38 cells in a dose-dependent manner, while TNFR2 conditioning further enhanced the capacity of nanoparticles in the reduction of viability (Fig. 3A and B; *P* < 0.001 to 0.05). Similarly, TNFR2 conditioning also resulted in markedly more cells undergoing apoptosis (12% for CT26 and 17.9% for MC38) as compared with ISO-PLGA-ADR nanoparticles (5.75% for CT26 and 9.79% for MC38) (Fig. 3C and D; *P* < 0.001). TNFR2-PLGA-ADR nanoparticle treatment resulted in the death of 26.38% of CT26 tumor cells and 29.59% of MC38 tumor cells (including early apoptosis, e.g., Annexin V^+^7AAD^−^ cells, and late apoptosis, e.g., Annexin V^+^7ADD^+^ cells), which was markedly higher than tumor cells treated with ISO-PLGA-ADR (20.15% and 24.54%, respectively; *P* < 0.01 to 0.05; Fig. S3). Thus, targeting TNFR2 markedly increased the in vitro cytotoxic effect of PLGA-ADR nanoparticles.

### In vivo distribution of nanoparticles with or without TNFR2 conditioning

**Fig. 4.**
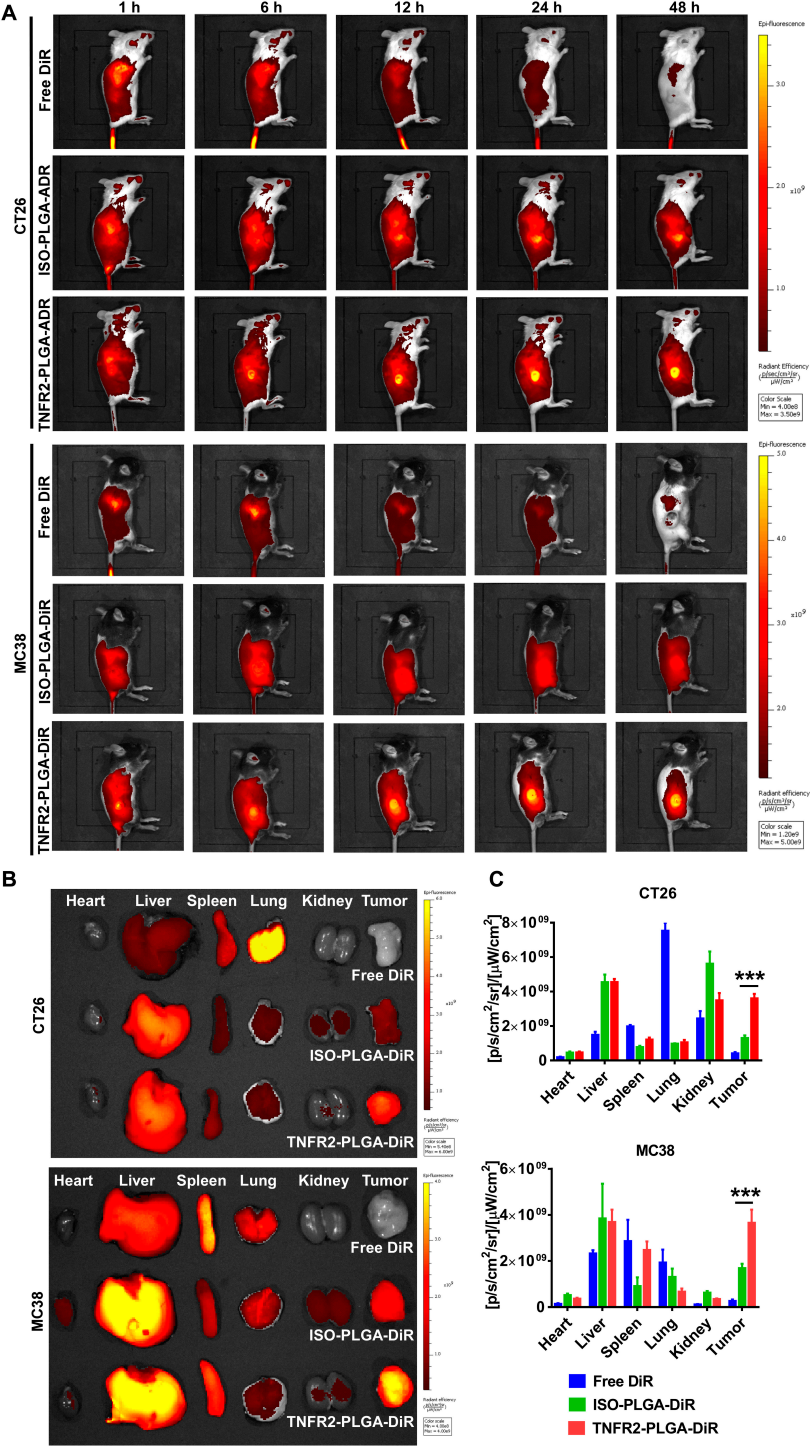
The distribution of ISO-PLGA-DiR and TNFR2-PLGA-DiR in mouse colon cancer. (A) Distribution of ISO-PLGA-DiR and TNFR2-PLGA-DiR. CT26 tumor-bearing BALB/c mice and MC38 tumor-bearing C57BL/6 mice were intravenously injected with fluorescent dye-labeled ISO-PLGA-DiR and TNFR2-PLGA-DiR as described in Materials and Methods when the volume of tumor reached 300 to 500 mm^3^, respectively. The distribution of ISO-PLGA-DiR and TNFR2-PLGA-DiR was examined and recorded with an in vivo imaging system for 1 to 48 h after injection. (B and C) Distribution of ISO-PLGA-DiR and TNFR2-PLGA-DiR in different organs. The mice were sacrificed 48 h after injection. The heart, liver, spleen, lungs, kidneys, and tumor were harvested, and the distribution of ISO-PLGA-DiR and TNFR2-PLGA-DiR was recorded using an in vivo imaging system (B). The average fluorescent intensity was quantified using the Maestro software (C). Data (mean ± SEM, *n* = 3) shown are representatives of 3 separate experiments with similar results. By comparison with ISO-PLGA-DiR, ***P < 0.001.

We next determined the distribution of TNFR2-targeted nanoparticles in mice bearing CT26 or MC38 tumors using an in vivo imaging system. For this purpose, TNFR2-PLGA and ISO-PLGA were labeled with 1,1′-dioctadecyl-3,3,3′,3′-tetramethyl indotricarbocyanine iodide (DiR) as tracer. Tumor-bearing mice were intravenously injected with free DiR or PLGA-DiR with or without TNFR2-targeting capacity. As shown in Fig. 4A, markedly higher levels of TNFR2-PLGA-DiR accumulated in the tumor tissues 24 h after injection, as evidenced by markedly stronger fluorescent signals. ISO-PLGA-DiR nanoparticles could also accumulate in the tumor tissue, presumably due to the passive targeting. However, its levels were significantly lower than that of TNFR2-PLGA-DiR (Fig. 4A). We also determined the levels of nanoparticles in different organs and tissues, including heart, liver, spleen, lungs, kidneys, and tumor, 48 h after injection. The results showed that the distribution of TNFR2-PLGA-DiR was markedly higher in the tumor tissues than its isotype control (Fig. 4B and C; *P* < 0.001). Consistent with a previous study [26], reticuloendothelial system (RES) in the lungs, liver, and spleen was able to retain high levels of DiR, while almost no free DiR signal could be detected in the tumor (Fig. 4B and C). Therefore, TNFR2-targeted nanoparticles had an enhanced capacity to permeate and accumulate in the tumor tissues.

### TNFR2 targeting markedly enhances the colocalization of ADR-encapsulated nanoparticles with T_regs_ in the tumor tissue

**Fig. 5.**
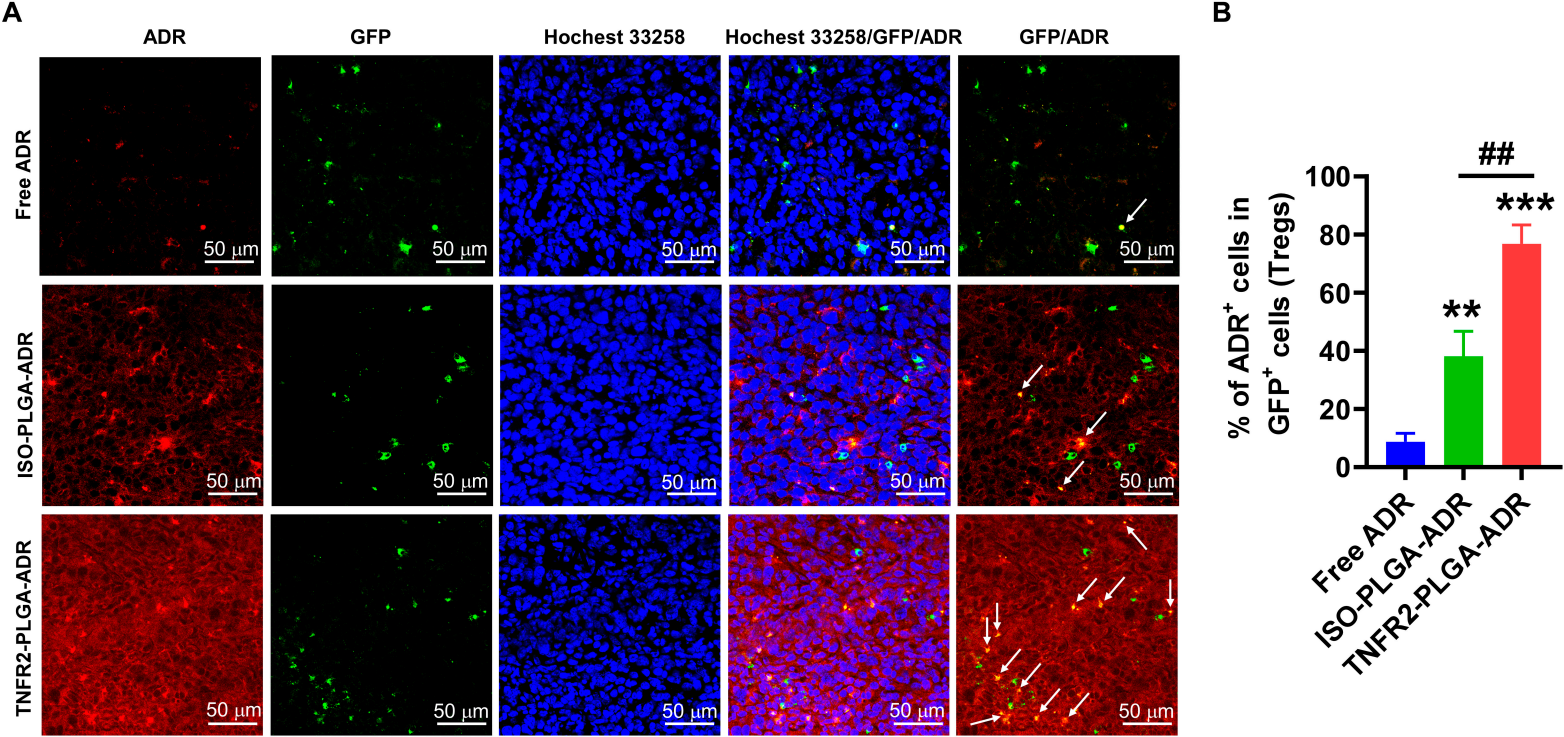
The colocalization of ISO-PLGA-ADR and TNFR2-PLGA-ADR with tumor-infiltrating T_regs_. Foxp3^EGFP/DTR^C57BL/6-Tg mice were inoculated in the right flank with MC38 cells (500,000 cells in 0.1 ml of PBS). When the size of tumor reached ~400 mm^3^, MC38 tumor-bearing Foxp3^EGFP/DTR^C57BL/6-Tg mice were intravenously injected with ADR, ISO-PLGA-ADR, or TNFR2-PLGA-ADR. After 24 h, the frozen section of different tumor tissue was stained with Hoechst 33258. (A) Levels of ADR (red) and EGFP (green, T_regs_) were measured using a confocal microscope (Zeiss 810). (B) Semiquantitative analysis for colocalization. Scale bar, 50 μm. Data shown are representatives of 3 separate experiments with similar results.

Since the majority of tumor-infiltrating T_regs_ are highly immunosuppressive TNFR2^+^ T_regs_ [13,27,28], we thus further examined if TNFR2-targeted nanoparticles could enhance the binding with tumor-infiltrating T_regs_. To this end, MC38 tumor-bearing Foxp3^EGFP/DTR^C57BL/6-Tg mice were intravenously injected with free ADR, ISO-PLGA-ADR, or TNFR2-PLGA-ADR. After 24 h, the tumor tissue cryosections were stained with Hoechst 33258, and the amount of ADR was recorded by CLSM. Results showed that the colocalization of TNFR2-PLGA-ADR with T_regs_ in the tumor tissues was markedly higher than that of ADR or ISO-PLGA-ADR (Fig. 5A and B; *P* < 0.001 to 0.01). Further, TNFR2-PLGA-ADR was dramatically more efficient in binding with tumor-infiltrating T_regs_ [green fluorescent protein (GFP); Fig. 5A and B; *P* < 0.001 to 0.01]. These data clearly showed that binding to T_regs_ contributed at least partially to the enrichment of TNFR2-PLGA-ADR in the tumor tissue. Nevertheless, we could not exclude the possibility that passive targeting may also play a role.

To further examine whether the anti-TNFR2 antibody-conjugated nanoparticles have active targeting capacity, CT26 tumor-bearing BALB/c mice or MC38-bearing C57BL/6 mice were established. When the tumor size was about 500 mm^3^ for the CT26 tumor or 300 mm^3^ for the MC38 tumor, the mice were injected with ADR, ISO-PLGA-ADR, or TNFR2-PLGA-ADR. The proportion of ADR^+^ cells in CD4^+^Foxp3^+^ T_regs_ in the spleen, liver, and tumor tissues was analyzed by FACS. The results show that the proportion of ADR-containing T_regs_ in the spleen and liver was quite low (0.5 to 6%), and there was no difference after TNFR2 conditioning (Fig. S4A to D). In contrast, the TNFR2-conditioned nanoparticles markedly enhanced the proportion of ADR-containing T_regs_ present in the tumor tissues (Fig. S4E and F; *P* < 0.05). Endothelial cells in the tumor could also express TNFR2 [18]; thus, we also examined if they were the cellular target of anti-TNFR2 antibody-conjugated ADR. The result showed that there was no binding of TNFR2-encapsulated nanoparticles to the endothelial cells (Fig. S4F and G). This is presumably because the expression of TNFR2 by endothelial cells was much less abundant than that of T_regs_ (Fig. S6). Taken together, these data favor the idea that the anti-TNFR2 antibody-conditioned nanoparticles were able to accumulate in the tumor tissues, and tumor-infiltrating TNFR2-expressing T_regs_ are a primary cellular target of this treatment.

### TNFR2-PLGA-ADR nanoparticles potently inhibit the growth of mouse colon cancers

**Fig. 6.**
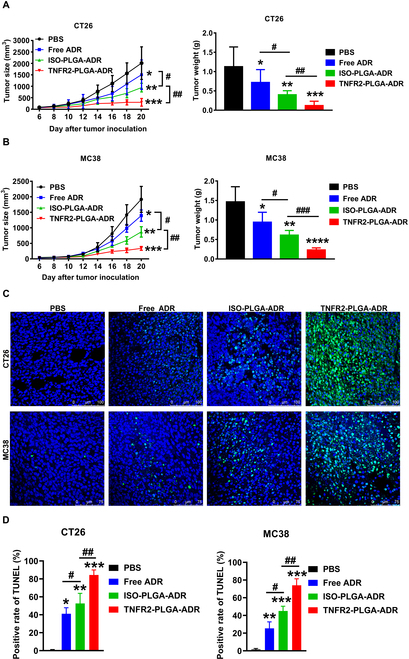
TNFR2-PLGA-ADR nanoparticles inhibit growth of mouse colon cancers. BALB/c mice were inoculated with CT26 cells (200,000 cells in 0.1 ml of PBS), and C57BL/6 mice were inoculated with MC38 cells (500,000 cells in 0.1 ml of PBS). On day 6 after tumor inoculation, CT26 tumor-bearing BALB/c mice and MC38 tumor-bearing C57BL/6 mice were randomly divided into 4 groups and intravenously injected with PBS, free ADR, ISO-PLGA-ADR, or TNFR2-PLGA-ADR every 3 d for 5 times, respectively. (A) The tumor size was monitored every other day. Tumor weight (B) was measured at the end of the experiment. Cell apoptosis (C) in the tumor tissue was evaluated by the TUNEL assay. Scale bars, 100 nm for CT26 tumor and 75 nm for MC38 tumor. (D) Semiquantitative analysis for cell apoptosis. The experiment was repeated for 3 times with similar results. Data shown are mean ± SEM (*n* = 6). By comparison with the PBS group, *P < 0.05, **P < 0.01, ***P < 0.001, ****P < 0.0001. By comparison with the indicated group, ^#^*P* < 0.05, ^##^*P* < 0.01, ^###^*P* < 0.001.

To examine the in vivo effect of TNFR2-targeted nanoparticles on tumor growth, tumor-bearing mice were intravenously injected with PBS, free ADR, ISO-PLGA-ADR, or TNFR2-PLGA-ADR for 5 dosages every 3 d, starting on day 6 after tumor inoculation. As shown in Fig. 6A and B, free ADR had just a moderate effect on the inhibition of tumor growth and reduction of tumor weight. ISO-PLGA-ADR showed a more potent antitumor effect than free ADR, while the antitumor effect of PLGA-ADR was markedly enhanced by the conjugation with anti-TNFR2 antibody (*P* < 0.0001 to 0.001). Tumor tissues were harvested after treatment, and apoptosis of tumor cells was analyzed by the terminal deoxynucleotidyl transferase–mediated deoxyuridine triphosphate nick end labeling (TUNEL) assay. As shown in Fig. 6C and D, free ADR only caused apoptosis of 39% (CT26) and 23% (MC38) of tumor cells (*P* < 0.01 to 0.05). ISO-PLGA-ADR treatment resulted in 55% (CT26) and 42% (MC38) of apoptosis cells in the tissues, which was markedly higher than that treated with free ADR (*P* < 0.001 to 0.01). Importantly, nanoparticles conditioned with anti-TNFR2 could cause a markedly higher number of apoptotic cells in the tumor (81% and 65%) as compared with that induced by the treatment with ISO-PLGA-ADR (Fig. 6C and D; *P* < 0.001). The difference of in vivo cytotoxicity between TNFR2-PLGA-ADR and ISO-PLGA-ADR (~20%) was higher than that observed in the in vitro study (~10%). It can be attributed to the targeting advantage of TNFR2-PLGA-ADR in the in vivo settings. Furthermore, once TNFR2-PLGA-ADR reaches the tumor site, TNFR2 antagonist antibodies can eliminate the immunosuppressive activity of T_regs_, acting with ADR synergistically to enhance the anticancer effect.

### The treatment with TNFR2-targeted nanoparticles reduces tumor-infiltrating TNFR2^+^ T_regs_ and increases IFNγ^+^CD8^+^ CTLs

**Fig. 7.**
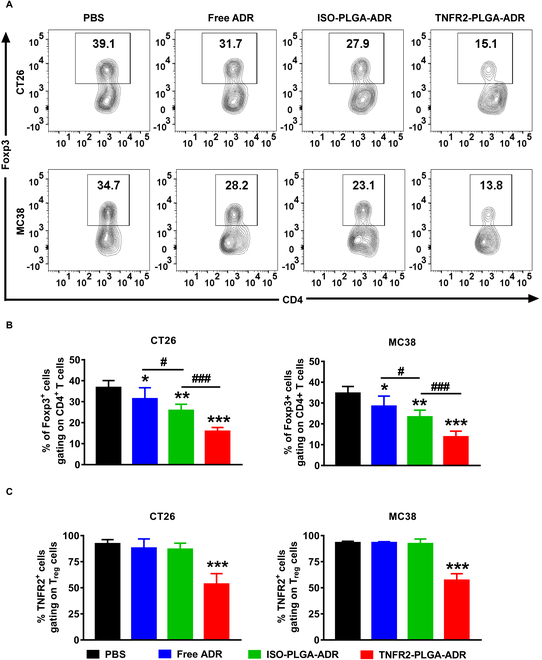
The in vivo effects of TNFR2-PLGA-ADR nanoparticles on T_regs_. CT26 tumor-bearing BALB/c mice and MC38 tumor-bearing C57BL/6 mice were treated as described in Fig. 6. (A to C) The proportion of T_regs_ in CD4^+^ cells and the expression of TNFR2 by T_regs_ were analyzed by FACS, gating for Foxp3^+^ cells. Representative FACS plots (A) and summary of proportion of T_regs_ (B). (C) Abundance of TNFR2 on the surface of T_regs_ in the tumor. The data (mean ± SEM, *n* = 6) shown in (B) and (C) are representative of 3 separate experiments with similar results. By comparison with the PBS group, *P < 0.05, **P < 0.01, ***P < 0.001. By comparison with the indicated group, ^#^*P* < 0.05, ^###^*P* < 0.001.

**Fig. 8.**
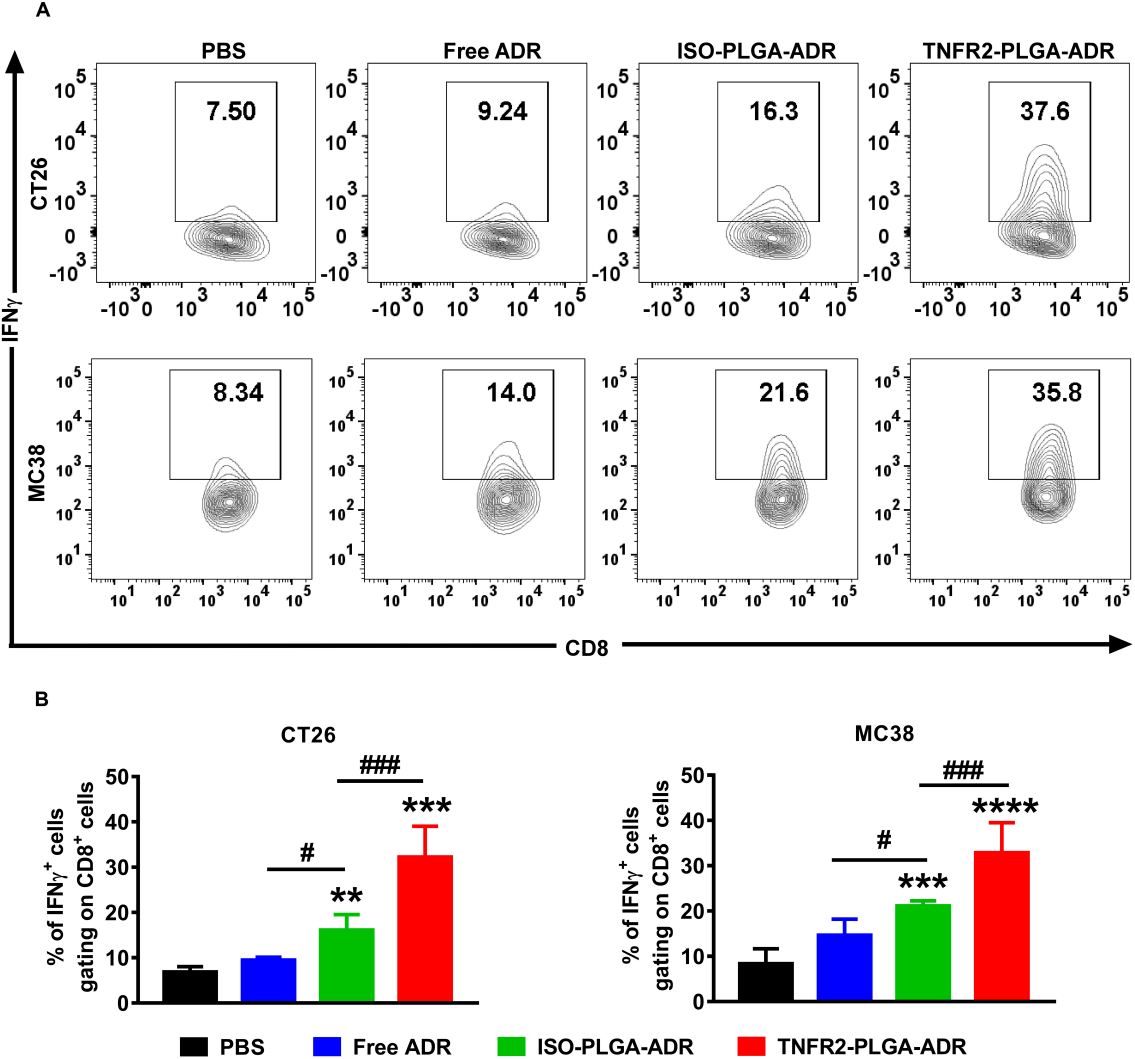
The in vivo effects of TNFR2-PLGA-ADR nanoparticles on tumor-infiltrating IFNγ^+^CD8^+^ CTLs. CT26 tumor-bearing BALB/c mice and MC38 tumor-bearing C57BL/6 mice were treated as described in Fig. 6. Single cells from tumor were restimulated in vitro, and intracellular IFNγ expression by CD8^+^ T cells was analyzed by FACS. Representative FACS plots (A) and summary (B). Bars (mean ± SEM, *n* = 6) shown are representative of 3 separate experiments with similar results. By comparison with the PBS group, *P < 0.05, **P < 0.01, ***P < 0.001. By comparison with the indicated group, ^#^*P* < 0.05, ^###^*P* < 0.001.

To investigate the effect of TNFR2-PLGA-ADR on tumor-infiltrating immune cells, we then examined the immune cell profile in the tumor microenvironment. As shown in Fig. 7A and B, the proportion of intratumoral T_regs_ was only reduced to 31.7% in CT26 tumor-bearing mice and 28.2% in MC38 tumor-bearing mice after the treatment with free ADR, as compared with PBS control (*P* < 0.05). Treatment with ISO-PLGA-ADR nanoparticles resulted in a more profound reduction of T_regs_ in the tumor (27.9% and 23.1% in CT26 or MC38 tumor-bearing mice; Fig. 7A and B; *P* < 0.01), while the TNFR2 expression on tumor-infiltrating T_regs_ remained unchanged (Fig. 7C). Interestingly, the TNFR2-PLGA-ADR treatment dramatically decreased intratumoral T_regs_ to 15.1% in CT26 tumor and 13.8% in MC38 tumor (Fig. 7A and B; *P* < 0.001), and the abundance of TNFR2 expression was markedly reduced as well (Fig. 7C; *P* < 0.001). Importantly, the TNFR2-PLGA-ADR nanoparticle treatment increased the expression of interferon γ (IFNγ) by CD8^+^ CTLs, and the proportion of IFNγ-producing CD8^+^ T cells was 2 to 3 times greater than free ADR treatment and 1.5 times greater than ISO-PLGA-ADR treatment (Fig. 8A to C; *P* < 0.001). Furthermore, mice treated with TNFR2-PLGA-ADR nanoparticles had higher serum IFNγ levels than other groups (Fig. S5; *P* < 0.01 to 0.05). Thus, our data indicated that TNFR2-PLGA-ADR nanoparticle treatment could reduce T_reg_ activity and promote the antitumor effect of CD8^+^ CTLs.

### Enhanced safety profile of TNFR2-PLGA-ADR nanoparticles

**Fig. 9.**
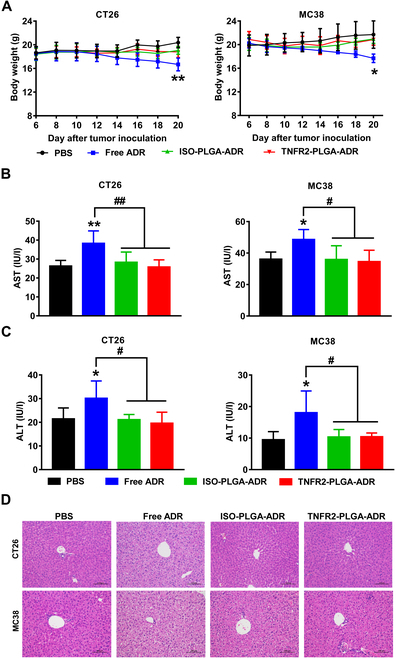
The systemic toxicity of TNFR2-PLGA-ADR nanoparticles in tumor-bearing mice. CT26 tumor-bearing BALB/c mice and MC38 tumor-bearing C57BL/6 mice were treated as described in Fig. 6. (A) Body weight of mice. (B and C) Forty-eight hours after the last injection, serum levels of AST (B) and ALT (C) were measured by using a commercial assay kit. (D) Histologic results of representative liver tissues from mice treated with PBS, ADR, ISO-PLGA-ADR, or TNFR2-PLGA-ADR (200×). Scale bar, 100 μm. Data (mean ± SEM, *n* = 6) shown are representative of 3 separate experiments with similar results. By comparison with the PBS group, *P < 0.05, **P < 0.01. By comparison with the indicated group, ^#^*P* < 0.05, ^##^*P* < 0.01.

The potential toxicity is the major concern of systemic chemotherapy. Indeed, the body weight of tumor-bearing mice was markedly reduced after the treatment with free ADR (Fig. 9A; *P* < 0.01 to 0.05) [29]. However, both ISO-PLGA-ADR- and TNFR2-PLGA-ADR-treated mice showed much less weight loss, suggesting alleviation of systemic toxicity of ADR by PLGA-PEG encapsulation. The potential toxicity of TNFR2-PLGA-ADR was further evaluated by measuring aspartate transaminase (AST) and alanine transaminase (ALT), the biochemical markers of liver injury, at 48 h after the last treatment. The histological alterations of the heart, liver, spleen, lungs, and kidneys were assessed by H&E staining. The results showed that the treatment with free ADR resulted in the elevation of AST and ALT (Fig. 9B and C; *P* < 0.01 to 0.05). Although pathological results of heart, spleen, lungs, and kidneys in all different treatment groups were no obvious lesion (Fig. S7), the pathological results of the liver in the free ADR-treated group showed significant lesion, including enlarged hepatocytes with microvesicular vacuolation and nuclear fragmentation (Fig. 9D). The encapsulation largely abolished this adverse effect of ADR with PLGA-PEG (e.g., ISO-PLGA-ADR or TNFR2-PLGA-ADR; Fig. 9B and C; *P* < 0.01 to 0.05). Therefore, PLGA-PEG encapsulation and modification with anti-TNFR2 antibodies (or isotype IgG antibodies) markedly reduce the systemic toxic effects and enhance the safety profile of ADR treatment.

## Discussion

The concept of targeted delivery has revolutionized the field of nanomedicine, providing hopeful approaches for accurate treatment and diagnosis [30]. Recent progress emphasizes the possibility of engineered nanoparticles that can selectively target diseased tissues, thereby enhancing therapeutic efficacy and decreasing adverse side effects [31–33]. As the field progresses, the integration of advanced targeting mechanisms with innovative nanoparticle designs is expected to result in more efficient treatment.

In the in vitro drug release system, an undesirable burst release of 43% ADR from nanoparticles was observed in the first 12 h (Fig. 1D). The uncontrolled initial burst release is common in most drug-loaded PLGA nanoparticles [34]. It is known that the main factors determining PLGA kinetics include particle size, porosity, and polymer molecular weight (MW), all of which are interrelated [35–37]. Fortunately, in our system, the preferential distribution of ISO-PLGA-DiR and TNFR2-PLGA-DiR in mouse colon cancer in a relatively short time frame is as follows: The nanoparticles started accumulating in the tumor site from 6 h after injection, and the number of accumulated nanoparticles increased over time (Fig. 4A). This rapid accumulation at the tumor site helps to offset the potential drawbacks of the initial burst release, ensuring effective drug delivery and therapeutic outcomes.

TNF receptor type I (TNFR1) and TNFR2 are 2 receptors that mediate the biological functions of TNF [38]. While TNFR1 is ubiquitously expressed on the surface of all types of cells except erythrocyte, TNFR2 expression is restricted to a few cell types [19]. Restricted cellular expression of TNFR2 makes it more attractive than TNFR1 as a molecular target for drug development [39]. We detected the expression of TNFR2 on different cells in the tumor tissues, and our results showed that although natural killer cells, dendritic cells, macrophages, and endothelial cells expressed TNFR2 to varying degrees, their abundance was far lower than that of T_regs_ (Fig. S6). Recently, the potential of anti-TNFR2 antibodies in cancer immunotherapy has been extensively investigated [18]. For example, murine-specific anti-TNFR2 antibody (TY101, 100 μg per mouse twice a week) was shown to have the capacity to increase the ratio of CD8^+^ T effectors (T_effs_) to T_regs_ by preferentially reducing TNFR2-expressing T_regs_ in the tumor microenvironment [40]. We reported recently that a combination of TNFR2 blocking antibody M861 (200 μg per mouse) and CpG oligodeoxynucleotides synergistically inhibited tumor growth in the mouse CT26 colon cancer model by reducing the TNFR2^+^ T_regs_ and increasing tumor-infiltrating IFNγ^+^CD8^+^ CTLs [41]. A dosage range of 100 to 200 μg of anti-TNFR2 was used for treating mouse tumor models [40,41], while there was no remarkable antitumor effect when suboptimal dose (20 μg) was administered [42]. In our study, a dose of TNFR2-PLGA-ADR comprises only 10 μg of anti-TNFR2 antibody. The results suggest that this dosage of anti-TNFR2 antibody was effectively improving the tumor-targeting capacities of PLGA-ADR. Nevertheless, it is of interest to explore the effect of TNFR2-PLGA treatment in mouse tumor models in subsequent study to examine if the potential of anti-TNFR2 antibody grafted on nanoparticles would exhibit greater potency in enhancing cancer-targeted delivery and promoting antitumor immune responses. In the current study, a marked antitumor effect was observed when the dosage of anti-TNFR2 antibody (BE0247) contained in nanoparticles was 10 μg per mouse for each injection. As shown by the TUNEL assay, the nanoparticle conditioned with anti-TNFR2 antibody possessed the potent activity in inducing apoptosis of cells in the tumor tissue (Fig. 6C). This antibody (BE0247) did not have the activity to induce the death of tumor cells in a concentration as high as 25 μg/ml, while PLGA-PEG was not toxic to tumor cells as well. Therefore, the potent antitumor effect of TNFR2-targeting nanoparticles is likely attributable to the preferential accumulation of ADR contained in TNFR2-PLGA-ADR in the tumor microenvironment and the target of TNFR2-expressing T_regs_ and cancer cells, while we could not exclude the EPR (enhanced permeability and retention) effect of nanodrugs. Furthermore, compared with ISO-PLGA-ADR nanoparticles, the treatment with TNFR2-PLGA-ADR nanoparticles markedly increased the IFNγ-expressing CD8 cells in the tumor (Fig. 8). Interestingly, a study published by the other group showed that the depletion of CD8 cells significantly reduced the mouse survival when treated with doxorubicin, while CD4 depletion did not, which demonstrated that the reduced tumor growth is CD8^+^ T cell dependent [43]. Thus, we favor the idea that the mobilization of CD8^+^ CTLs is attributable to the effect of TNFR2-PLGA-ADR nanoparticles in mouse tumor models.

As previously documented, CT26 and MC38 colon cancer cells exhibit high levels of TNFR2 expression [23]. In this study, the FACS and CLSM results demonstrated that anti-TNFR2 antibody enhances the binding affinity of PLGA-ADR to tumor cells. In vivo distribution results further confirmed the role of TNFR2 in the targeting specificity of TNFR2-PLGA-ADR. It was repeatedly reported that TNFR2 expression on tumor-infiltrating T_regs_ is much higher than that on splenic and lymph node T_regs_ [13]. Confocal microscopy data from this study clearly show the direct interaction of TNFR2-PLGA-ADR nanoparticles and TNFR2-expressing T_regs_ in the tumor microenvironment (Fig. 5). Additionally, ADR levels in tumor-infiltrating T_regs_ were markedly higher in mice treated with TNFR2-PLGA-ADR compared with splenic T_regs_, liver T_regs_, and tumor-infiltrating endothelial cells (Fig. S4E and F). Furthermore, we detect TNFR2 expression on different cell types in the tumor microenvironment and found that the TNFR2 levels on these cells were substantially lower than that on T_regs_ (Fig. S6). Collectively, our current data strongly support the notion that TNFR2-expressing tumor cells and tumor-infiltrating TNFR2-expressing T_regs_ are primary cellular targets of TNFR2-PLGA-ADR nanoparticles. This explains why TNFR2-PLGA-ADR nanoparticles could preferentially accumulate at tumor sites.

Although the treatment with TNFR2-PLGA-ADR markedly reduced TNFR2-expressing tumor-infiltrating T_regs_, TNFR2 expression and proportion of T_regs_ in the spleen were not inhibited (data not shown). Presumably, the discrepancy is caused by the much lower levels of TNFR2 expression by T_regs_ present in the spleen, as compared to that expressed by T_regs_ in the tumor [13]. Since the levels of TNFR2 expression are associated with immunosuppressive function of T_regs_ [13], it is predictable that TNFR2-PLGA-ADR treatment may markedly reduce the function and amount of T_regs_. Unfortunately, we have no chance to examine immunosuppressive activity of tumor-infiltrating T_regs_ in mice treated with TNFR2-PLGA-ADR, due to the paucity of T_regs_ isolated from tumor tissues. Although endothelial cells have been reported to express TNFR2, we examined the amount of ADR in tumor-infiltrating endothelial cells, and the results showed that the proportion of TNFR2 antibody-modified nanoparticles taken up by endothelial cells was not significantly different from that of the free ADR group and ISO-PLGA-ADR nanoparticle groups. This may be due to the fact that endothelial cells, despite expressing TNFR2, are not abundant and are much lower than tumor-infiltrating T_regs_. In the tumor microenvironment, other types of immunosuppressive cells, such as myeloid-derived suppressor cells (MDSCs), also express TNFR2 [18]. These TNFR2-expressing MDSCs are likely also to be the target of TNFR2-PLGA-ADR treatment, and this possibility will be examined in future study.

Chemotherapeutic agent such as ADR, by inducing tumor cell death, remains a predominant treatment in the clinic and an object of current interest of research [44]. Although effective in the treatment of cancer patients in the clinic, the systemic administration of ADR could cause severe adverse effects, including multi-organ toxicity, which remains to be the major obstacle for its clinical application [45]. It was shown that normal mice treated with ADR could also result in heart toxicity, hepatotoxicity, and nephrotoxicity [46], and these harmful adverse effects of ADR were confirmed in our study. Importantly, the treatments with nanoparticle encapsulated ADR had no obvious toxicity to heart, spleen, lungs, and kidneys (Fig. S7). Moreover, encapsulation with PLGA-PEG nanoparticles (both ISO-PLGA-ADR and TNFR2-PLGA-ADR) almost completely abolished the detrimental impact of ADR on hepatotoxicity (Fig. 9). Therefore, encapsulation with PLGA-PEG nanoparticles can markedly enhance the safety profile of ADR and thus hold promise in the clinical application. Intravenously delivered nanoparticle drugs typically undergo 5 consecutive processes [47]: circulation within the blood compartment, accumulation in the target area, subsequent penetration into the tissue, cellular uptake, and intracellular release of the drug from endosomes or lysosomes. Lysosomes contain various degradative enzymes, such as nucleases and phosphatases, and the inability to rapidly escape from lysosomes usually results in encapsulation and potential degradation, leading to reduced efficiency of therapeutic drug delivery [48]. Therefore, the ability to escape from endosomes/lysosomes is one of the key indicators for evaluating or improving TNFR2-PLGA-ADR systems in the future [49].

## Materials and Methods

### Mice, cells, and reagents

Female BALB/c, male C57BL/6 mice, and Foxp3^EGFP/DTR^C57BL/6-Tg mice, 8 to 12 weeks old, were initially purchased from Jackson Laboratory and maintained under specific pathogen-free conditions in the Animal Facility of the University of Macau. The animal research protocol was approved by the Animal Research Ethics Committee of the University of Macau (protocol ID: UMARE-018-2023).

The BALB/c-derived CT26 colon cancer cell line was purchased from the American Type Culture Collection (ATCC). CT26 colon cancer cells were cultured in RPMI 1640 medium supplemented with 10% FBS and 2 mM glutamine at 37°C and 5% CO_2_ in a humidified incubator. The MC38 colon cancer cell line was purchased from iCell Bioscience Inc. (Shanghai, China). MC38 colon cancer cells were cultured in Dulbecco’s modified Eagle’s medium (DMEM) supplemented with 10% FBS and 2 mM glutamine at 37°C and 5% CO_2_ in a humidified incubator.

Antibodies used in this study, including PerCP-Cy5.5 anti-mouse TCRβ (H57-597), allophycocyanin (APC) anti-mouse CD8a (53-6.7), phycoerythrin (PE) anti-mouse IFNγ (XMG1.2), Pacific Blue anti-mouse CD4 (RM4-5), and PE anti-mouse CD120b (TNFR2, TR75-89), were purchased from BD Pharmingen (San Diego, CA). PE-Cy7 anti-mouse CD4 (GK1.5), fluorescein isothiocyanate (FITC) anti-mouse CD45 (30-F11), and APC anti-mouse/rat Foxp3 (FJK-16s) were purchased from Invitrogen (eBioscience). LIVE/DEAD Fixable Near-IR Dead Cell Stain Kit and TRlzol Reagents were purchased from Thermo Fisher Scientific. The Annexin V-APC/7-AAD apoptosis kit was purchased from MultiSciences Biotech Co. Ltd. (Hangzhou, China). InVivoMAb anti-mouse TNFR2 (CD120b, BE0247) and InVivoMAb polyclonal Armenian hamster IgG (isotype control, BE0091) were purchased from BioXCell. mPEG2k-PLGA5k (PLGA, copolymer ratio 50:50) and NHS-PEG2k-PLGA5k (PLGA, copolymer ratio 50:50) were purchased from Shanghai Mao Kang Biotechnology Co. Ltd. (Shanghai, China). ADR (A183027) was purchased from Aladdin (Shanghai, China). High-performance liquid chromatography (HPLC)-grade tetrahydrofuran and methanol were purchased from Merck (Darmstadt, Germany). Hematoxylin and Eosin Staining Kit was purchased from Beyotime Institute of Biotechnology (Jiangsu, China). DiR and Hoechst 33258 were purchased from Invitrogen (CA, USA). AST and ALT detection kits were purchased from Nanjing Jiancheng Institute of Biotechnology (Nanjing, China).

### Preparation of nanoparticles

ADR-loaded PLGA-PEG-NHS (PLGA-ADR) was prepared using a modified procedure of oil-in-water single-emulsion solvent evaporation. ADR was dissolved in methanol at the concentration of 2 mg/ml. PLGA-PEG-NHS was dissolved in tetrahydrofuran at the concentration of 80 mg/ml. When completely dissolved, ADR and PLGA-PEG-NHS were mixed at a 1:1 volume ratio and sonicated for 2 min as the organic phase. To obtain nanoparticle suspension, the mixed solution (organic phase) was quickly injected into an aqueous solution containing 6.25% methanol under the condition of intense ultrasound. The organic mixture (including tetrahydrofuran and methanol) was removed rapidly by evaporation under nitrogen gas at 37°C. After centrifuging at 4,000 rpm in a 10,000 MW ultrafiltration tube for 20 min and washing twice by PBS, the unencapsulated ADR was removed. The supernatant was filtered with a 0.45-μm filter to obtain ADR-loaded (PLGA-ADR) nanoparticle solution. PLGA-ADR nanoparticles conjugated with anti-TNFR2 antibody (or isotype IgG antibody) were prepared as previously reported [50]. Briefly, the TNFR2 antibody (or isotype IgG antibody) solution was mixed with PLGA-ADR nanoparticle solution at the molar ratio of 1:4 in PBS. After 6 h, TNFR2-PLGA-ADR and ISO-PLGA-ADR were purified with a Sephacryl S-300 column (Amersham Biosciences). A BCA kit was used to measure the amount of anti-TNFR2 antibody (or isotype control antibody) conjugated to the PLGA-ADR surface [51,52].

### Characterization of PLGA-ADR, ISO-PLGA-ADR, and TNFR2-PLGA-ADR nanoparticles

The particle size, PDI, and ζ potential of PLGA-ADR, ISO-PLGA-ADR, and TNFR2-PLGA-ADR were determined by DLS (Nano-Zetasizer, Malvern, UK) at 25°C. The surface morphology of these nanoparticles was characterized by using scanning electron microscopy. Scanning electron microscopy tests were performed by using Zeiss Zigma FESEM and an x-ray energy spectrometer. Before testing, the dried samples were coated with a thin layer of gold to improve its electrical conductivity. The test voltage was 5 kV. To determine drug (ADR) LC of TNFR2-PLGA-ADR and ISO-PLGA-ADR, freeze-dried TNFR2-PLGA-ADR and ISO-PLGA-ADR were dissolved in dimethyl sulfoxide (DMSO), and the amount of ADR was determined using the fluorescent spectrophotometer. The EE and LC of ADR by PLGA-ADR were calculated using the following equations: EE = Drug in nanoparticles/Total drug added information × 100%, LC = Weight of loaded ADR/Weight of PLGA-ADR × 100%.

### In vitro drug release behavior of ISO-PLGA-ADR and TNFR2-PLGA-ADR nanoparticles

To mimic the drug release in vivo, the drug release behavior of ISO-PLGA-ADR and TNFR2-PLGA-ADR was studied by dialysis. Briefly, 3 ml of the prepared ISO-PLGA-ADR or TNFR2-PLGA-ADR solution (equivalent ADR dose of 0.5 mg/ml) was transferred into a dialysis bag (MW cutoff, 3,500) and immersed in 15 ml of pH 7.4 PBS at 37°C. The medium was harvested at different times as indicated, and an equal amount of fresh medium was added. The release of ADR was measured quantitatively by an ultraviolet (UV) spectrophotometer.

### Cellular binding and subcellular distribution

The binding of ADR and antibody-conjugated nanoparticles by CT26 and MC38 mouse colon cancer cells was examined by flow cytometry and CLSM. The cells were first inoculated in 24-well plates with a density of 1 × 10^5^ per well and incubated overnight. Then, CT26 and MC38 cells were incubated at 37°C for 4 h with free ADR, ISO-PLGA-ADR, TNFR2-PLGA-ADR, or TNFR2-PLGA-ADR plus anti-TNFR2 antibody (25 μg/ml) pretreated for 1 h (equivalent ADR concentration of 1 μg/ml). The cells without any treatment were used as the control. After 4 h of treatment, the supernatant was removed and 1 ml of 4°C PBS solution was added to stop cellular binding and to wash the cells. Then, 200 μl of trypsin-EDTA was added to digest the cells for 1 min. After that, 0.4 ml of complete medium was added to stop the digestion, and the cells were harvested and centrifuged at 1,000 rpm for 5 min. After discarding the supernatant, 500 μl of PBS was added to disperse the cells in a uniform single-cell suspension. The fluorescence intensity of ADR in 10,000 cells from each sample was measured by flow cytometry. Based on the flow cytometry results, CLSM was used to visually study the binding of ADR and antibody-conjugated nanoparticles to CT26 and MC38 colon cancer cells as follows: The cells were seeded on an 8-well Lab-Tek chambered cover glass (Thermo Fisher Scientific) at a density of 2.5 × 10^4^ cells per well and cultured at 37°C overnight. After the cells were well attached, they were treated at 37°C for 4 h with free ADR, ISO-PLGA-ADR, TNFR2-PLGA-ADR, or TNFR2-PLGA-ADR plus anti-TNFR2 antibody (25 μg/ml) pretreated for 1 h (equivalent ADR concentration of 1 μg/ml). After washing with PBS for 3 times, the cells were fixed with 4% paraformaldehyde for 15 min and then rinsed with PBS for 3 times, followed by staining with Hoechst 33258 (Invitrogen, USA) for 5 min. Then, the cells were washed for 3 times with PBS and covered with 0.3 ml of PBS. The amount of ADR (red) was determined by using CLSM (TCS SP8II, Leica, Germany).

### In vitro cell cytotoxicity and apoptosis assays

CT26 and MC38 mouse colon cancer cells in a logarithmic growth phase were seeded in 96-well plates at a density of 6,000 per well. After incubation for overnight, the culture medium was removed, and 100 μl of new medium containing ISO-PLGA-ADR or TNFR2-PLGA-ADR nanoparticles at equivalent ADR concentrations was added. The control group was cultured with medium without any nanoparticles. After incubation for 24 h, 20 μl of MTT [3-(4,5-dimethylthiazol-2-yl)-2,5-diphenyltetrazolium bromide] solution (5 mg/ml) was added. Four hours later, 100 μl of DMSO was added to each well, and the purple crystals were sufficiently dissolved by shaking for 15 min. The optical density was measured at 570 nm by using a microplate reader (Molecular Devices, USA). The proportion of cell viability (% of control) was calculated.

To determine the apoptosis, cells were treated as aforementioned for 24 h. Then, the cells were collected in precooled PBS. After washing, the cells were resuspended in 500 μl of 1× binding buffer and 5 μl of Annexin V-APC, and 10 μl of 7-AAD was added to each tube. After gentle vortexing, the cells were incubated at room temperature for 15 min in the dark. Apoptosis of cells was analyzed by using the BD LSRFortessa flow cytometer.

### Mouse tumor models and treatment

Eight-week-old female BALB/c or C57BL/6 mice were subcutaneously injected with 200,000 CT26 cells or 500,000 MC38 cells at the right flank, respectively. On day 6 after tumor inoculation, the mice were randomly divided into 4 groups and intravenously injected with PBS, ADR (2 mg/kg), ISO-PLGA-ADR, or TNFR2-PLGA-ADR nanoparticles (containing 2 mg/kg of ADR) every 3 d for 5 dosages. The tumor volume and mouse body weight were monitored every other day, and tumor size was calculated using the following formula: volume = 0.5 × (width)^2^ × (length).

### In vivo biodistribution of nanoparticles

CT26 tumor-bearing BALB/c mice or MC38 tumor-bearing C57BL/6 mice with a tumor size of around 300 to 500 mm^3^ were intravenously injected with free DiR, ISO-PLGA-DiR, or TNFR2-PLGA-DiR (0.015 mg DiR per mouse), respectively. The in vivo fluorescent signals were recorded at 1, 6, 12, 24, and 48 h after injection by using the in vivo imaging system (CRi Maestro, Woburn, MA). Mice were sacrificed at 48 h after injection, and the organs, including heart, liver, spleen, lungs, kidneys, and tumor, were removed for fluorescent imaging. The average fluorescence intensities were calculated by the following formula: average signals (counts/s/pixel) = total counts/exposure time (s)/area (pixel).

To determine cell accumulation of nanoparticles to the T_regs_ of the tumor microenvironment, Foxp3^EGFP/DTR^C57BL/6-Tg mice, bearing MC38 tumors with a size of around 400 mm^3^, were intravenously injected with free ADR, ISO-PLGA-ADR, or TNFR2-PLGA-ADR (0.1 mg of ADR per mouse). After 24 h, the tumor cryosection was stained with Hoechst 33258 and imaged by using a confocal microscope (Zeiss 810).

To determine the binding of nanoparticles to the T_regs_ in spleen and liver and endothelial cells in the tumor, MC38 tumor-bearing C57BL/6 mice with a tumor size of around 300 mm^3^ or CT26 tumor-bearing BALB/c mice with a tumor size of around 500 mm^3^ were intravenously injected with free ADR, ISO-PLGA-ADR, or TNFR2-PLGA-ADR (0.1 mg of ADR per mouse). After 48 h, spleens, livers, and tumors were excised, minced, and digested in RPMI 1640 supplemented with collagenase IV (1 mg/ml) and deoxyribonuclease I (0.1 mg/ml) at 37°C for 1 h. The fragments were pushed through a 70-μm pore size cell strainer to create a single-cell suspension. The proportion of ADR^+^ cells gating on T_regs_ or endothelial cells (CD31^+^) was determined by a flow cytometer.

### Intracellular cytokine staining

For detection of CD4^+^Foxp3^+^ T_regs_ in the tumor tissue, tumors were excised, minced, and digested in RPMI 1640 supplemented with collagenase IV (1 mg/ml) and deoxyribonuclease I (0.1 mg/ml) at 37°C for 1 h. The fragments were pushed through a 70-μm pore size cell strainer to create a single-cell suspension. The cells were stained with a Live/Dead cell stain kit. Then, the cells were fixed and permeabilized with Cytofix/Cytoperm (Invitrogen, USA) and incubated with appropriately diluted anti-CD45, anti-TCRβ, anti-CD4, anti-Foxp3, and anti-TNFR2 antibodies after blocking the Fc receptor. The proportion of Foxp3^+^ cells in live CD45^+^CD4^+^ T cells, as well as TNFR2 expression by T_regs_, was determined by flow cytometry. For detection of IFNγ^+^CD8^+^ CTL cells in the tumor tissue, the single cells obtained in the above manner were restimulated for 6 h with phorbol 12-myristate 13-acetate (20 ng/ml) and ionomycin (1 μM) in the presence of GolgiPlug in a 96-well plate. The cells were stained with a Live/Dead cell stain kit. Then, the cells were fixed and permeabilized with Cytofix/Cytoperm (Invitrogen, USA) and incubated with appropriately diluted anti-CD45, anti-TCRβ, anti-CD8, and anti-IFNγ antibodies after blocking the Fc receptor. The proportion of CD8^+^ T cells in live CD45^+^CD4^+^ T cells, as well as expression of IFNγ in CD8^+^ T cells, was determined by flow cytometry. The acquisition was performed with a BD LSRFortessa flow cytometer. The FlowJo software was used in the analysis of FACS data. The gating strategy for tumor-infiltrating T_regs_ (CD4^+^Foxp3^+^) is shown in Fig. S1.

### TUNEL assay

TUNEL staining kit (C1091) was purchased from Beyotime (Shanghai, China). The tissue slices of mouse tumors were first treated with 20 μg/ml of proteinase K (ST532, Beyotime) and further incubated with immune wash buffer (P0106, Beyotime) for 20 min at 20°C. After incubation with sealing solution (P0100B, Beyotime) for 20 min at room temperature, the tissue slices were further incubated with 0.05 ml of marker buffer for 60 min at 37°C in the dark. Then, the stop buffer was added and further incubated for 10 min at room temperature. After that, the tissue slices were incubated with streptavidin–horseradish peroxidase solution for 30 min at room temperature and the 0.3-ml DAB (3,3′-diaminobenzidine) buffer for 20 min. Then, the tissue slices were washed with PBS for 3 times. The tissue slices were observed and documented by using CLSM (TCS SP8II, Leica, Germany).

### Biosafety evaluation

Tumor-bearing mice were intravenously injected with PBS, ADR (2.0 mg/kg), ISO-PLGA-ADR, or TNFR2-PLGA-ADR nanoparticles (containing 2.0 mg/kg of ADR) for 5 dosages as described above. According to the manufacturer’s instruction, serum levels of AST and ALT were determined at 24 h after the last injection by using commercial detecting kits. Twenty-four hours after the last injection, the mice were sacrificed, and organs (heart, liver, spleen, lungs, and kidneys) were collected and fixed in 4% paraformaldehyde for overnight, followed by dehydration with gradient alcohol solutions, and then embedded in paraffin. The fixed tissues were cut into 4-μm sections and stained with hematoxylin and eosin (H&E) to assess the histological alterations by using an inverted microscope.

### Statistical analysis

All data were presented as means ± SEM, and the statistical analysis was performed by *t* test or one-way analysis of variance (ANOVA) test by using GraphPad Prism 7.0. A *P* value of <0.05 was considered to be statistically significant.

## Data Availability

All data needed to evaluate the conclusions of the study are present in the paper and the Supplementary Materials. Additional data related to this paper may be requested from the author upon reasonable request.
