# Supplementary figures and images for: Anti-TNFR2 Antibody-Conjugated PLGA Nanoparticles for Targeted Delivery of Adriamycin in Mouse Colon Cancer
Source: Research (Wash D C). 2024 Sep 6;7:0444. doi: 10.34133/research.0444 (PMC11377996; doi:10.34133/research.0444)

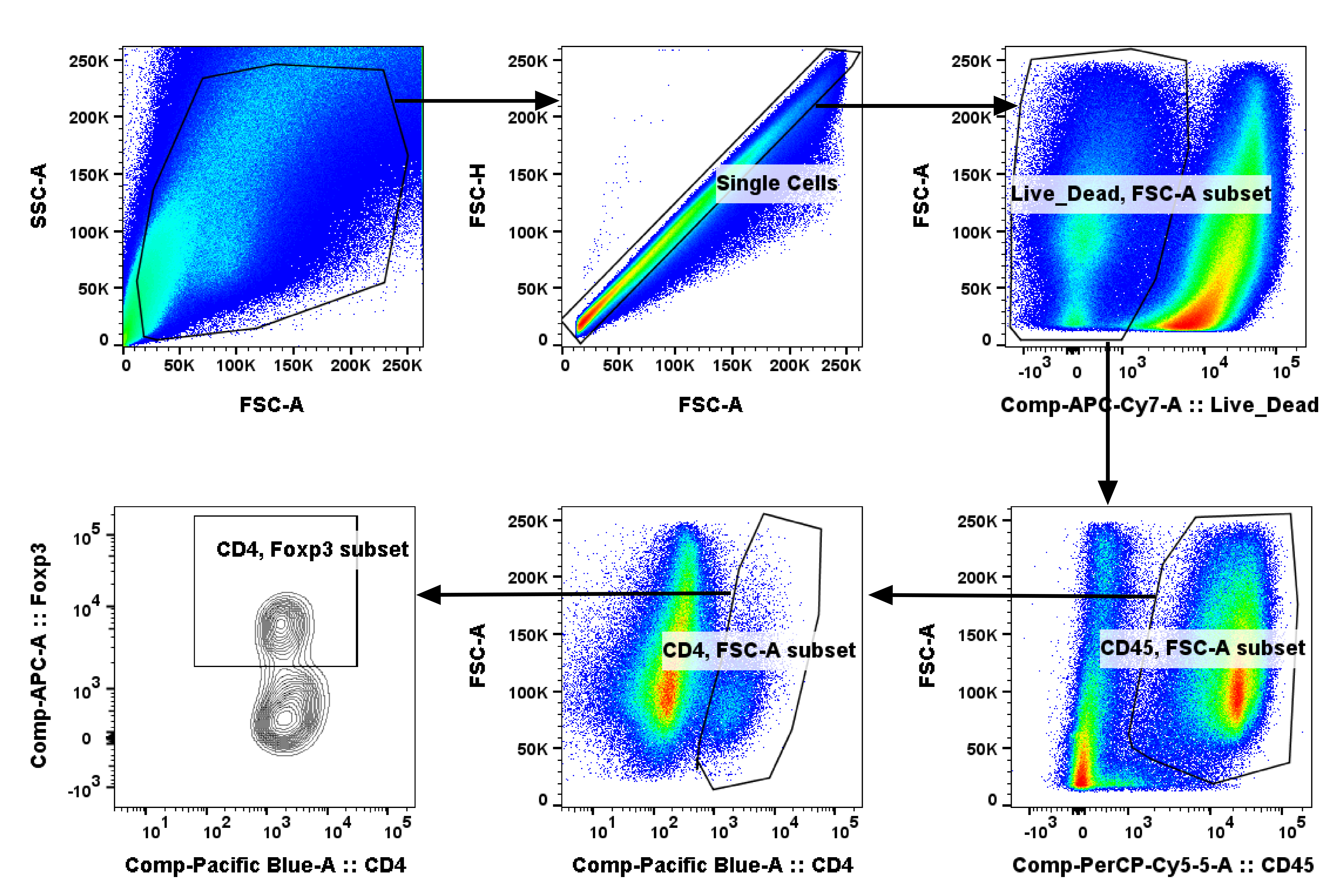

Supplement: Supplementary 1 — Figs. S1 to S7 [file research.0444.f1.zip › Figure S1.tif]

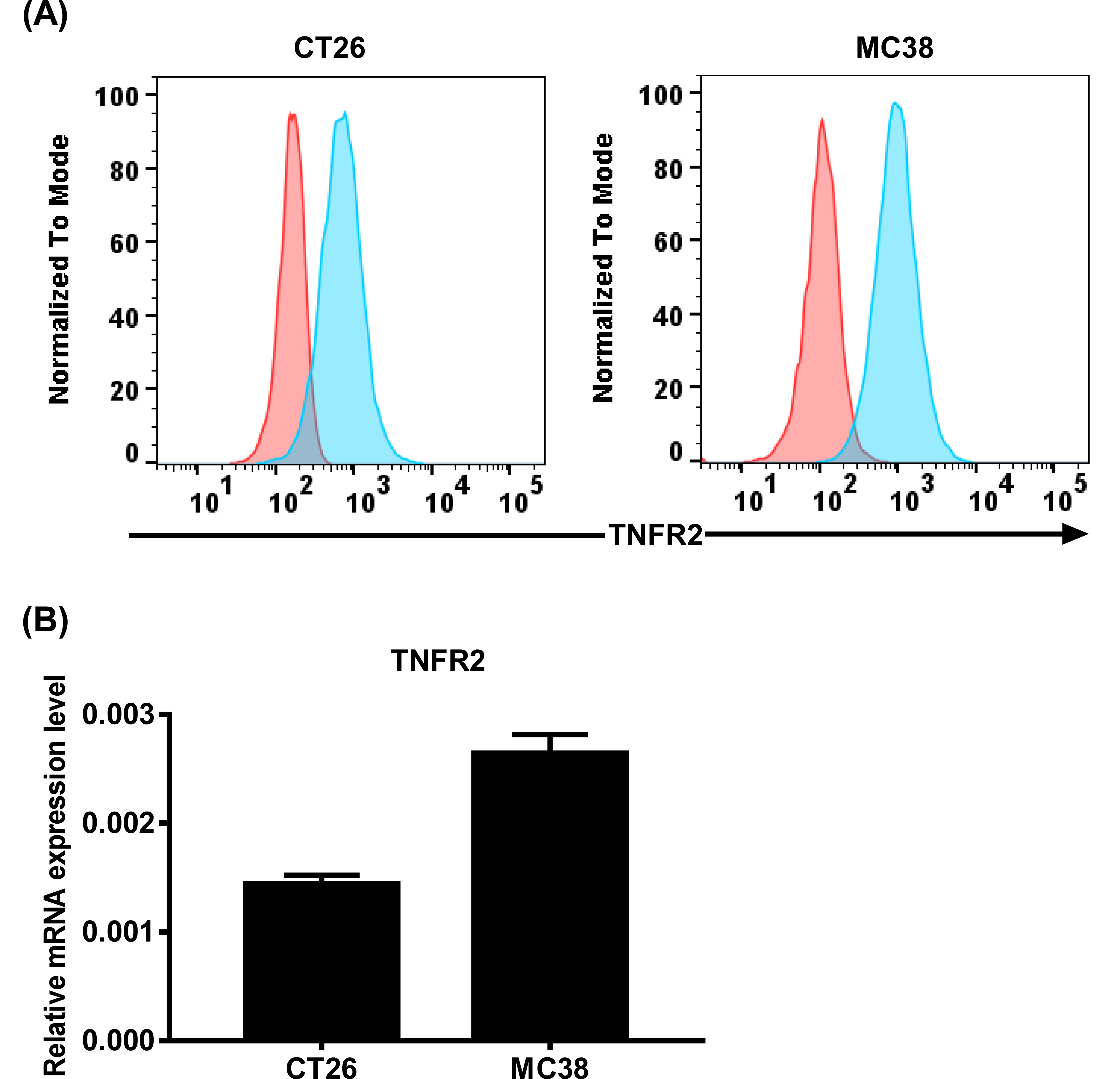

Supplement: Supplementary 1 — Figs. S1 to S7 [file research.0444.f1.zip › Figure S2.tif]

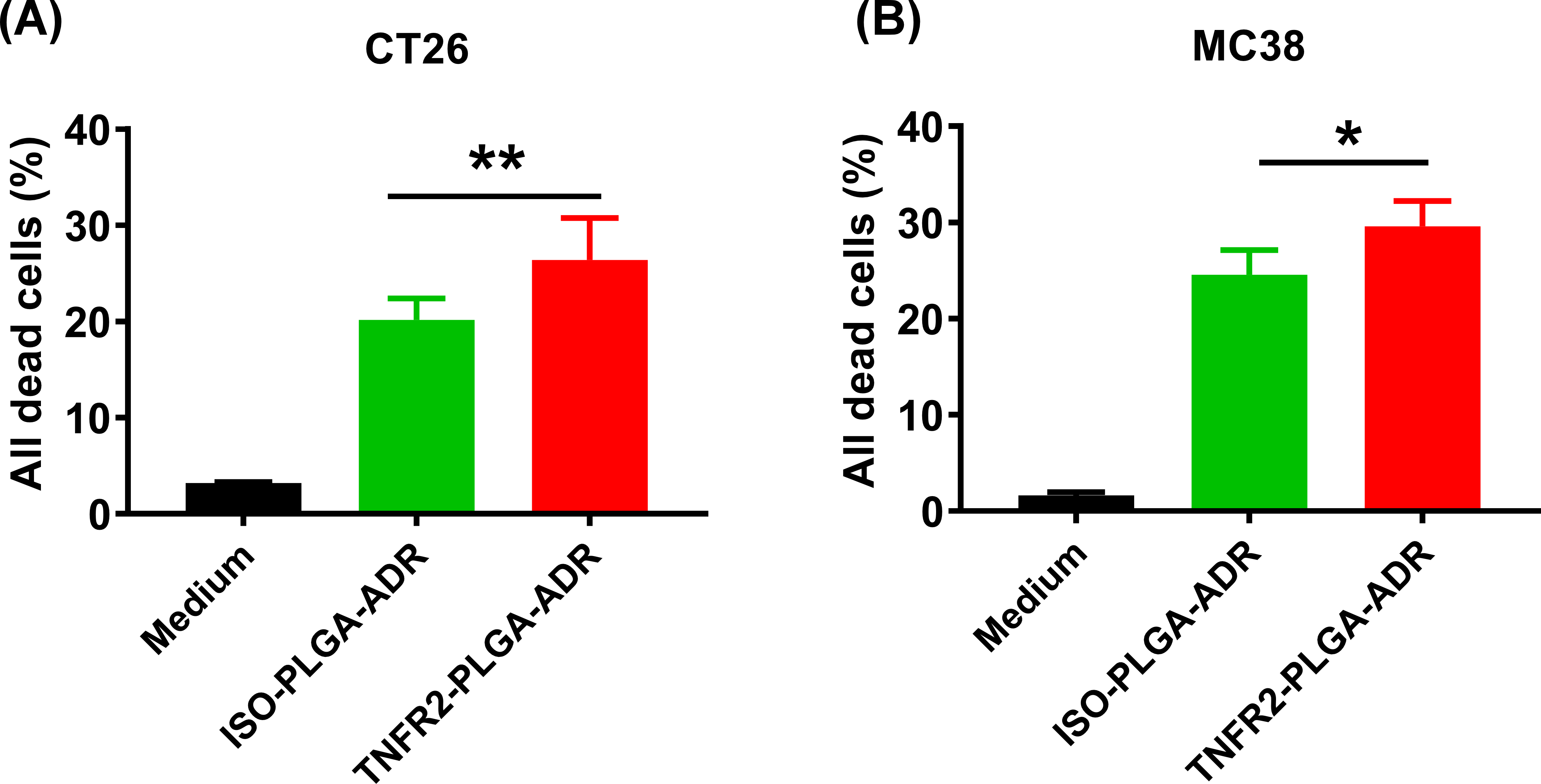

Supplement: Supplementary 1 — Figs. S1 to S7 [file research.0444.f1.zip › Figure S3.tif]

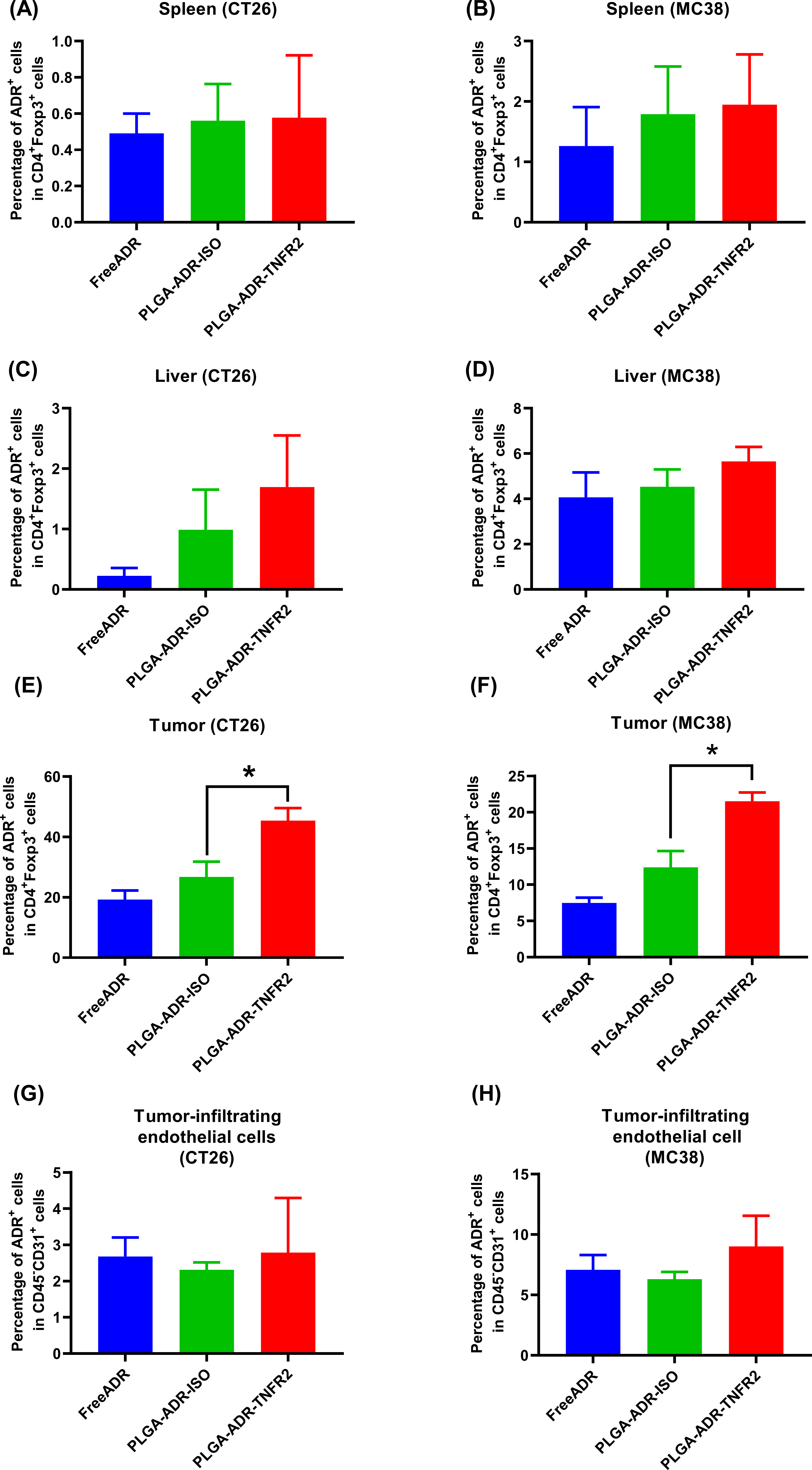

Supplement: Supplementary 1 — Figs. S1 to S7 [file research.0444.f1.zip › Figure S4.tif]

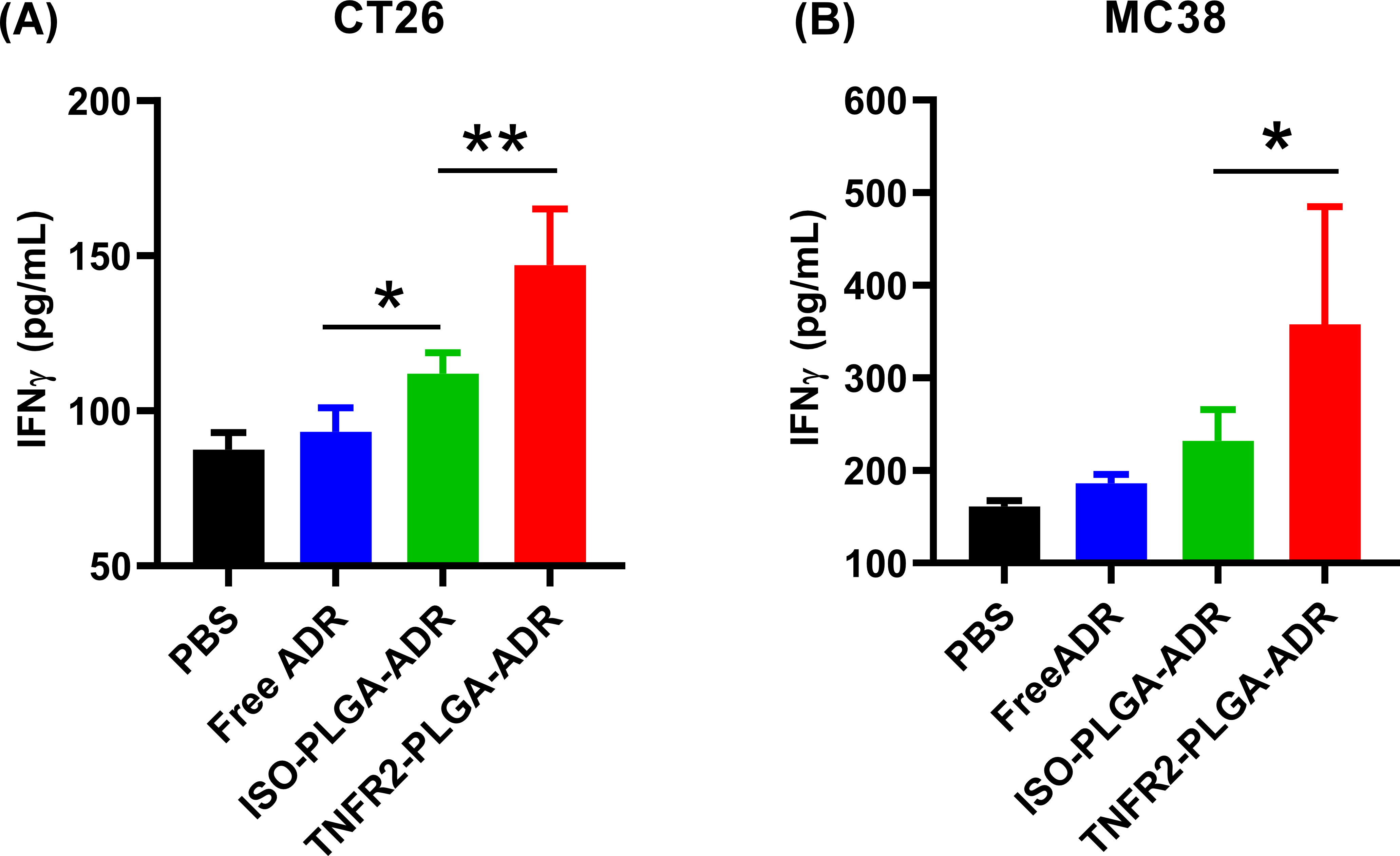

Supplement: Supplementary 1 — Figs. S1 to S7 [file research.0444.f1.zip › Figure S5.tif]

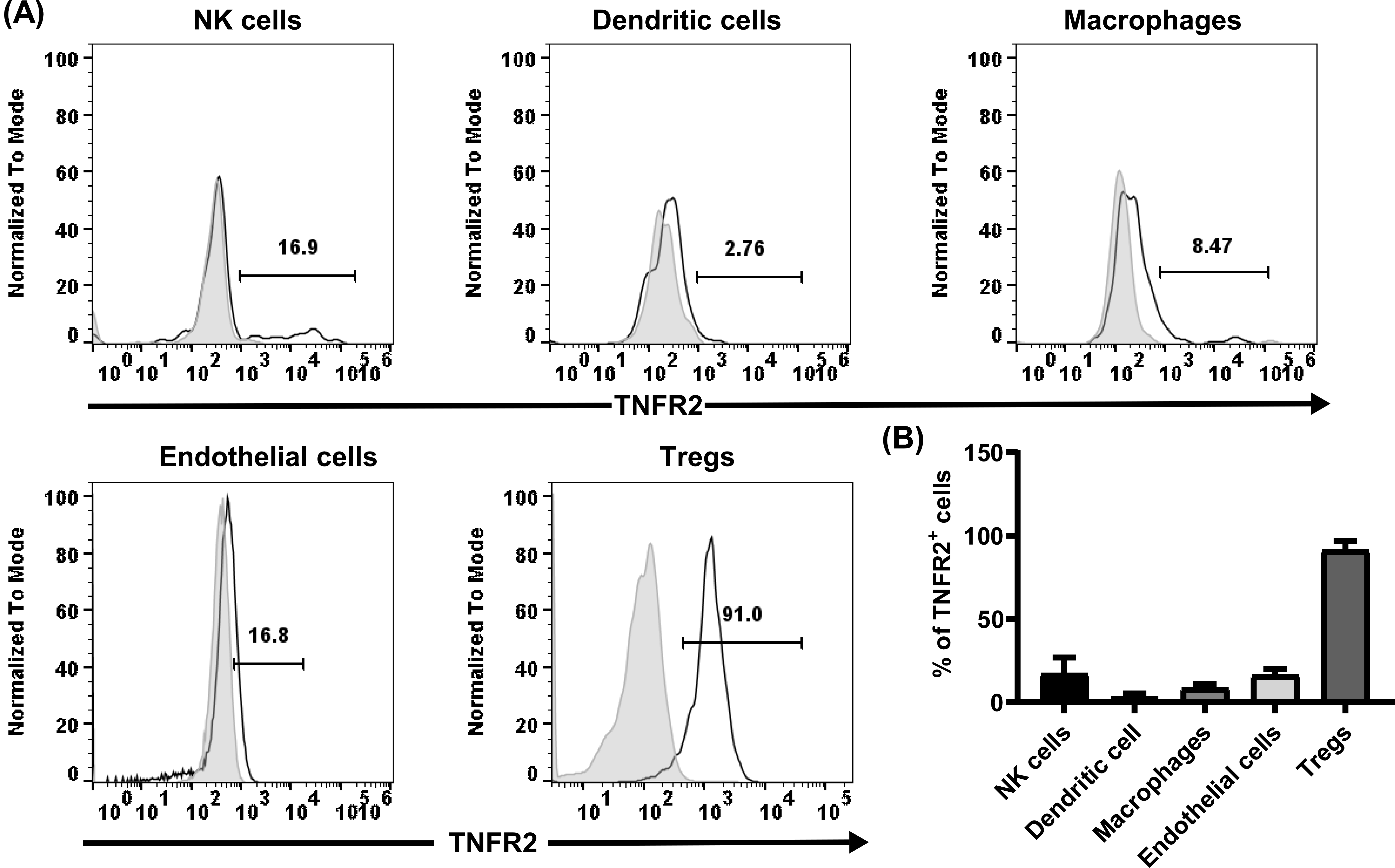

Supplement: Supplementary 1 — Figs. S1 to S7 [file research.0444.f1.zip › Figure S6.tif]

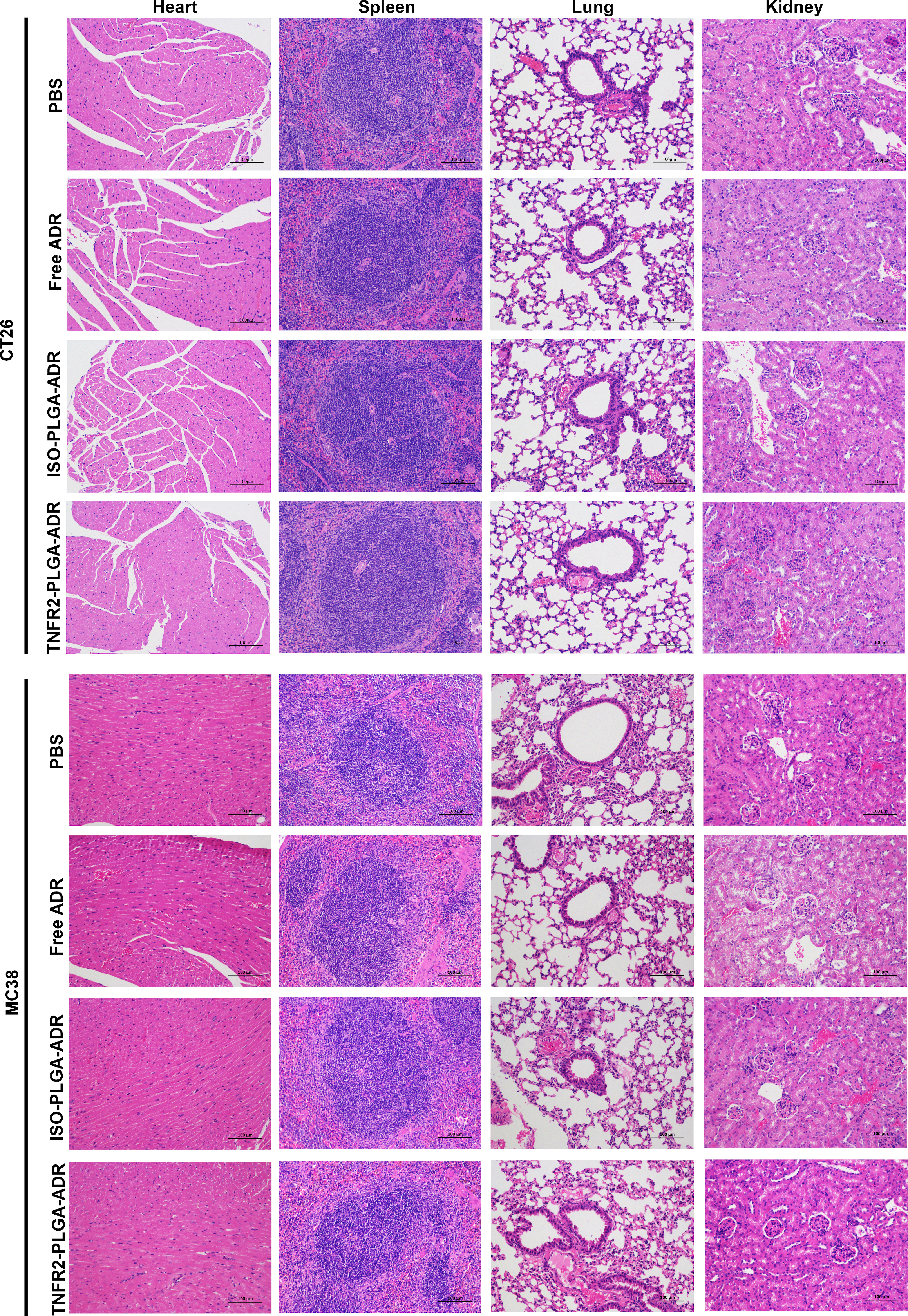

Supplement: Supplementary 1 — Figs. S1 to S7 [file research.0444.f1.zip › Figure S7.tif]
